# A South African Perspective on the Microbiological and Chemical Quality of Meat: Plausible Public Health Implications

**DOI:** 10.3390/microorganisms11102484

**Published:** 2023-10-03

**Authors:** Christy E. Manyi-Loh, Ryk Lues

**Affiliations:** Centre of Applied Food Sustainability and Biotechnology, Central University of Technology, Bloemfontein 9301, South Africa; rlues@cut.ac.za

**Keywords:** sustainable animal farming, meat, bacterial pathogens, antibiotic resistance, public health, South Africa

## Abstract

Meat comprises proteins, fats, vitamins, and trace elements, essential nutrients for the growth and development of the body. The increased demand for meat necessitates the use of antibiotics in intensive farming to sustain and raise productivity. However, the high water activity, the neutral pH, and the high protein content of meat create a favourable milieu for the growth and the persistence of bacteria. Meat serves as a portal for the spread of foodborne diseases. This occurs because of contamination. This review presents information on animal farming in South Africa, the microbial and chemical contamination of meat, and the consequential effects on public health. In South Africa, the sales of meat can be operated both formally and informally. Meat becomes exposed to contamination with different categories of microbes, originating from varying sources during preparation, processing, packaging, storage, and serving to consumers. Apparently, meat harbours diverse pathogenic microorganisms and antibiotic residues alongside the occurrence of drug resistance in zoonotic pathogens, due to the improper use of antibiotics during farming. Different findings obtained across the country showed variations in prevalence of bacteria and multidrug-resistant bacteria studied, which could be explained by the differences in the manufacturer practices, handling processes from producers to consumers, and the success of the hygienic measures employed during production. Furthermore, variation in the socioeconomic and political factors and differences in bacterial strains, geographical area, time, climatic factors, etc. could be responsible for the discrepancy in the level of antibiotic resistance between the provinces. Bacteria identified in meat including *Escherichia coli*, *Listeria monocytogenes*, *Staphylococcus aureus*, *Campylobacter* spp., *Salmonella* spp., etc. are incriminated as pathogenic agents causing serious infections in human and their drug-resistant counterparts can cause prolonged infection plus long hospital stays, increased mortality and morbidity as well as huge socioeconomic burden and even death. Therefore, uncooked meat or improperly cooked meat consumed by the population serves as a risk to human health.

## 1. Introduction

Meat is the word used to describe the flesh, skeletal muscles, or any attached connective tissue or fat without bone and bone marrow. Meat consumption is experiencing a global increase; however, its consumption shows disparities from country to country, and this is strongly linked to disposable income [1]. The general increase in meat consumption often provokes intensification of the production process either via improved technology or application of chemicals and equipment that will assist in preventing disease transmission amongst the animal population in a bid to ensure a rise in productivity [2]. According to Milford et al. [1], South Africa is amongst the countries projected to contribute to the total increase in meat consumption, because of the rising urbanisation, elevated income, and rapid population growth.

Therefore, the country adopts intensive farming to address the heightened demand in meat and its products. The increased consumption of meat products offers an increased risk of exposure to pathogens of animal origin [3]. These meats are consumed as unprocessed meats or processed meats; however, Papier and colleagues [4] demonstrated that meat consumption is associated with higher risks of many disease conditions. In addition, intense production will usually culminate in increases in animal wastes, huge consumption of antibiotics, overcrowding on farms, and increases in greenhouse gas emissions (climate change), causing environmental degradation as well as the release of antibiotics and antibiotic resistance genes into soil and water, which in turn promotes the development and the dispersal of pathogens resistant to antibiotics plus their resistance genes (ecological effects) [5,6]. It is for these reasons that Mathur and co-authors [7] advised that meat consumption should be reduced in order to better human health, curtail environmental degradation and greenhouse gas emissions, and reduce the large-scale suffering of animals living on factory farms. Moreover, taking into consideration the listed parameters in South Africa, including 28% of adults with obesity and the presence of food insecurity and diet-related diseases associated with high intake of meat, it is ideal to explore a more healthy, sustainable, and equitable protein supply [2].

There are both formal and informal sectors dealing with meat in South Africa, as meat and its products can be purchased from butcheries, retail shops, selected farms, and open markets. In addition, meat handled in the formal sector is liable to several checks to confirm its safety, based on microbial level and composition, before release to the consumers. However, meat discharged from informal outlets is not subjected to such routine checks, therefore, there is a great likelihood of contamination resulting in poor meat quality and safety. The formal meat sector is more regulated, and the meat produced is projected to be of improved quality, relating to microorganism level, than meat produced by the counterpart sector, wherein issues of a lack of hygiene, poor sanitation, and poor meat quality have been reported [8]. Despite the challenges observed in the informal meat sector, Kalule and co-authors [9] noted that the informal abattoirs, including the streetside slaughter of livestock and the sales of meat and meat products, remain as a vital part of the microeconomy. 

Food contamination can occur by way of food handlers, food-producing animals, food contact surfaces, food-processing tools or equipment, dust, and air [10]. Contamination of raw meat by microorganisms can occur during processing at different stages, including slaughtering, scalding, dressing, evisceration, cutting, distribution, and storage [11,12]. The application of appropriate temperatures on raw meat during processing (cooking) can kill the vegetative forms of some bacteria, but their enterotoxins evade the processes involving thermal treatments because they are thermostable and can equally demonstrate resistance to gastrointestinal proteases [13]. In relation to its composition, fresh meat is viewed as a highly perishable foodstuff. 

Although foods are contaminated by naturally occurring pathogenic microorganisms, food safety is a crucial issue because safe food is a basic right of every individual (human) in the world [14]. In addition, South Africa enacted the Meat Safety Act (Act 40 of 2000) with sections 51 and 52 focusing on poultry regulations whilst sections 53 and 54 pertain to regulations involving red meat [15]. The authors mentioned further that the Meat Safety Act consists of measures to provide safety of meat and animal products and define the phrase “unsafe for animal and human ingestion” as meat that is unsafe owing to a disease condition, an abnormal condition, decomposition, putrefaction, contamination, or residues or because of exposure or contact with decomposed, diseased, or putrefied or contaminated material.

Meat and meat-based products are the common major reservoirs for microorganisms, among the food types investigated [16]. Meat consists of different portions of proteins (19%), fat (2.5%), and carbohydrates (1.2%), a great fraction of water in the muscle cells (75%), and nitrogenous compounds (1.65%) [17]. Hence, it contains a good source of numerous nutrients and presents as an appropriate medium for the growth of microorganisms due to its water activity and optimum pH [18]. The measure of contamination by microbes and microbial composition of meat mirrors its hygienic condition. The provision of meat with microbial counts not above the microbial contamination limit is expected to fulfil the requirements for achieving safe, healthy, and wholesome meat maximising the shelf life [19]. In this light, the Meat Safety Act in South Africa guides the abattoirs that deliver the meat to retailers for purchase by the consumers with a scope of protocols and regulations ensuring that the meat produced is of high quality and safe for consumption, with a minimal likelihood of microbial contamination [20]. However, meat inspection at the abattoir involves visual inspection without any microbiological assessment, and the distribution chain from the abattoirs to the retail outlets could be easily compromised, leading to contamination [21]. However, the microbiological quality of meat after slaughter depends largely on the type of meat, processing, distribution, and storage conditions (temperature, oxygen demand, pH, and competing organisms) [22]. 

Microbiological analysis is an established tool in monitoring the safety and quality of meat and its products [23]. Apparently, the microbiological status of the meat gives an indication of the quality and safety of the meat. It can be expressed as viable counts of bacteria in the mass of the meat tested or comprises a highly variable microbial community. Based on the quantity of the bacteria represented as colony-forming units per gram of meat or abundance or the level of microbial diversity, the meat may be defined as contaminated [15]. Of high risk is the consumption of processed meat products that are termed as ready-to-eat foods (e.g., polony) as they require no further preparation [24]. Pathogenic bacteria of zoonotic origin that have been incriminated in the outbreak of meat-related foodborne diseases include *Moraxella* spp., *Salmonella*, *Listeria monocytogenes*, *E*. *coli*, *Clostridium perfringens*, *Bacillus cereus*, *Staphylococcus aureus*, etc., associated with wastes of meat products, causing great economic losses that affect the domestic market and international trade [22]. In addition, human consumption of meat contaminated with microbes results in public health implications, causing huge illnesses or even deaths [24]. Nevertheless, foodborne infections/diseases are more prevalent in immunocompromised individuals plus children [25].

Of brutal consequences are cases that involve the antibiotic-resistant counterparts of the previously mentioned bacteria as well as their resistance genes as antibiotic resistance genes are disseminated amongst bacteria through horizontal gene transfer. Jaja and colleagues [26] demonstrated a variation in the proportions of antimicrobial resistance of meat between the formal and informal meat sector and ascribed the discrepancy to the sample size, the management systems involved in enforcing hygiene, the use of antibiotics for treatment or disease prevention in the animals, and the treatment of entire herds without selecting animals with no infections in both sectors. Therefore, food serves as a significant vehicle for transmission of pathogenic microorganisms together with their toxins and antibiotic-resistant bacteria to humans, causing diseases and infections. This seems to be a very serious and potential challenge in low-income countries [11].

This paper assembles knowledge of animal farming (poultry and livestock), the number of ways meat and its products can be contaminated, whether microbial or chemical (antibiotics), in addition to the mechanisms of generation and dissemination of antibiotic resistance in animal farming as well as the ways of controlling resistant pathogens. Lastly, findings on the prevalence of multidrug resistance in foodborne pathogens occurring in the different provinces of the country have been gathered and the public health implications of consuming contaminated meat are outlined. 

## 2. Animal Farming and Associated Sources of Contamination (Microbial and Chemical Contamination)

Owing to factors such as health, economy, and culture, meat and meat products represent a bigger part of typical human food enjoyed by the population across the world. Apparently, the South African meat industry contributes to food security, the nutritional wellbeing of the population, in addition to the growth of the economy [27]. The country is observing a high intake of meat and this behaviour triggers meat production, which is a human activity [6]. According to Taljaard et al. [28], the demand for meat by the South African population is influenced by a host of factors, viz., disposable income, the price of meat in itself, changes in the size and the structure of the population, meat price related to other products as well as changes in the taste and preferences of the consumers. Approximately, 2.9 million tons of meat (poultry, pork, and beef) is consumed by South Africans per year, and more than 60% of the total meat consumption is poultry meat [29]. The authors further mentioned that the production of beef, pork, and poultry is 2.4 million tons and is complemented by imports from Brazil, the Netherlands, the United Kingdom, Germany, and Argentina. In general, the meat types include chicken, beef, pork, lamb, and mutton. Meat from cattle and poultry serves as the main source of protein for subsistence groups living in several African countries, therefore, they depend on meat production for their livelihood. It is either processed or left unprocessed and the meats can be sold raw or prepared in public restaurants for sale as ready-to-eat meats [4].

### 2.1. Types of Animal Farming

#### 2.1.1. Poultry Farming

Poultry farming describes the domestication of birds, including local (indigenous) and commercial chickens (broilers), geese, turkeys, ducks, quails, guinea fowls, and pigeons [30]. It is usually performed by small-scale farmers and large-scale commercial farmers to produce eggs and meat; chickens farmed for egg production are known as layers while broilers are chickens farmed for meat [31]. Usually, production in poultry farming takes less than six weeks and, consequently, it is one of the world’s largest sources of meat [32]. 

Viljoen [32] mentioned that South Africa produces the greatest fraction of chicken in Africa, producing approximately 2152 million tonnes of poultry meat consumed per year by the population of the country. AGRIFARMING [33] remarked that the subtropical climate of the country makes it ideal for farming with plants, livestock, and poultry. The South African Poultry Association (SAPA) [34] encompasses both the commercial and smallholder farmers within the day-old chick supply, broiler, and egg industries. The largest fraction of the agricultural sector in South Africa is poultry farming, contributing greatly to the GDP. North West Province is amongst the provinces noted with the largest production and distribution of broilers [35] and the companies RCL and Astral are the largest producers in the country [30]. Owing to efficient feed conversion, chickens can acquire the highest growth rates and lowest cost per unit output, giving them an advantage over other livestock that can do this to a similar extent [36]. A great rise in chicken consumption has been demonstrated in the country [37]. To ensure microbial quality of the poultry cuts during storage, vacuum packaging, chilling, and marinades are amongst the methods employed and are determined by the habits of the consumers and the country in question [38]. According to Esterhuizen [29], poultry meat is the most important protein source served in the diets of most South Africans since it is inexpensive and ubiquitous. 

In Africa, including South Africa, intensive, semi-intensive, and extensive systems are the components of poultry farming; intensive farming is performed by large-scale and commercial farmers in the form of deep litter systems for broilers and battery cages for layers, wherein scheduled feeds are administered [39]. In addition, huge application of antimicrobials occurs in this practice. In semi-intensive practice, the birds are permitted to scavenge during the day over a well-demarcated and fenced area while, at night, they are kept in houses and administered feeds. Free range and backyard practices are the aspects of extensive systems usually applied by small-scale and household farmers [30]. As the name connotes, in free range practice, the birds are allowed to stray freely over an extended land area and elementary shelters may be available, but the birds may scavenge outside. Meanwhile, in backyard farming, the poultry are allowed to scavenge, but this practice is supplemented with the administration of feed, and at night the birds are kept in their houses. Notwithstanding, in extensive and semi-intensive farming, there is proximity between the birds and humans; a scenario of significant concern to public health due to the likelihood of hazards [40]. 

In developing countries, including South Africa, poor rural households and urban areas depend on poultry farming as the main source of livelihood, therefore, these farming practices vary from the city to local areas in the provinces of South Africa. For example, Eastern Cape Province is the poorest amongst all the provinces in South Africa and the inhabitants depend on natural subsistence for livelihood [41]. Therefore, animal faeces (poultry) are used as fertiliser in agricultural fields or as feed for fish, without adequate treatment, which is a potentially risky practice from the environmental and public health perspectives [40].

One of the economically significant agroindustries is poultry farming, but it experiences economic losses owing to the high mortality rate and decreased productivity rate due to diseases caused by *Salmonella* species, which are amongst the causative agents of infections in poultry and avian species [42]. These organisms are important in animal farming in terms of the emergence of antibiotic resistance in the strains. In the life cycle of chickens, *Salmonella* spp. can be vertically transmitted from the infected parents to chicks or via horizontal transmission through hatcheries, contaminated feed, and equipment, demonstrating sex in contaminated hatcheries, and cloacal infection [43]. After slaughtering of chickens, the meat can be sold as whole chickens, chicken breasts, anus, hearts, gizzards, kidneys, necks, legs, wings, and livers, and might be processed into sausages.

#### 2.1.2. Livestock Farming (Cattle, Pig, Goat, Sheep)

The South African market for meat is affected by the growing economy and population in conjunction to the emerging black middle class, however, almost all the consumers in the country are very sensitive to price relating to beef purchases [44]. The authors further commented that meat is the most rapidly growing agricultural commodity worldwide and in the meat industry, the procedures, including slaughtering, processing, and the preservation of the meat, are amongst the value-added activities taking place and creating job opportunities. Globally, pork production and consumption lead, however, this is not the case with South Africa, as the population does not express the same love of pork at all. This is ascribed to a heterogeneous consumer population with changing needs and preferences but, particularly, different races and cultural and religious groups give pork a wide berth [44]. Nevertheless, pork remains as a good, affordable source of red meat in comparison to beef and mutton [45]. Notwithstanding, DAFF [46] mentioned that South Africa experienced an increase in pork consumption from 3.9 kg to 4.7 kg (20.5%) from 2005 to 2015 via a consumer education/promotion initiative sponsored by statutory levy income. Lubinga et al. [27] highlighted that the consumers are educated on the health and nutritional advantages of pork and its products and are assured of a safe product because of a quality assurance and traceability scheme. The authors emphasised that pork is a nutrient-dense food, containing plenty of essential nutrients, viz., vitamins, minerals, and protein; the protein is described as complete and highly digestible since it contains all the necessary amino acids.

In particular, provinces including North West, Gauteng, Limpopo, and Mpumalanga are described as the largest producers of pork in South Africa. Predominantly, no less than five (5) breeds are produced for commercial purposes (SA Landrace, Large White, Duroc, Pietrain, and Kolbrook) and pig carcasses are produced either as porkers or baconers. The porkers (60 kg) can be differentiated from baconers (between 70 and 100 kg) in terms of weight and use; porkers are utilised as fresh meat, while baconers are further processed in the meat industry into other meat products, including sausages, polonies, meat rolls and spread, bacon, hams, and Russians. Furthermore, the country has embraced an ever-expanding beef industry and KwaZulu-Natal Province is the second most prominent cattle producer, contributing significantly to the supply of beef in the country [47]. The country also practices communal farming, wherein the livestock are put in separate stalls at night but allowed to graze together on pasture, occurring in the communities [48].

Ground beef is the most popular amongst the varieties of beef products sold and, owing to its versatile nature and low price, every average individual within the population can consume it, thus it constitutes 60% of all retail beef sales. However, ground beef is highly vulnerable to microbial contamination due to its process of production as the grinding process causes an increase in the surface area of the beef and, consequently, a greater part of the meat becomes exposed to bacteria [49]. Notwithstanding the precautions considered, meat often harbours a high concentration of microbes even in instances where hormones and antibiotics have been employed [50]. Accordingly, the Food and Agriculture Organization (FAO) and World Health Organization (WHO) have established limits or thresholds for microbiological estimation or the bacterial load that is considered safe for human consumption [11].

In response to the Meat Safety Act (Act 40 of 2000), the owner of an abattoir is expected to reveal a list that entails all the possible hazards that might occur from biological sources, with subsequent management programmes relying on hygiene to avert, eradicate, or lessen the recognised hazards to satisfactory points. According to the Codex Alimentarius Commission [51], many standards, codes of practice, strategies, and other references concerning food, food manufacturing, and safety of food have been developed under Codex Alimentarius. The slaughtered bodies of pigs and cattle can be dissected and sold as meat in various forms, including sausages, ground beef, beef stew, beef chunks, pork, ribs, legs, skin, head, tripes (internal organs), tripes hearts, and livers. Surprisingly, contamination still occurs within the food value chain despite the efforts presented, since it is a complicated process [15]. Contamination of meat and its products readily occurs through several food hazards, comprising biological, chemical, physical, and especially microbial factors. 

### 2.2. Types of Contamination

#### 2.2.1. Microbial Contamination of Meat

The most important zoonotic foodborne pathogens recovered from meat include *Escherichia coli*, *Salmonella* spp., *Aeromonas hydrophila*, *Campylobacter jejuni, Staphylococcus aureus*, *Listeria monocytogenes*, *Clostridium perfringens*, and *Yersinia enterocolitica* [11,18]. Nevertheless, inconsistencies do exist in the data collected on the prevalence of bacterial species identified in meat investigated in different developing countries as shown in Table 1.

Owing to the lack of numerous preservation barriers, storage facilities, rapid detection assays, and lack of understanding of the growth mechanism of microorganisms, particularly the dearth of knowledge about intricate bacterial foodborne pathogens, the risk of occurrence of foodborne pathogens in South Africa is high [52]. Remarkably, contamination via animal faeces stands as the primary source of contamination and could extend to the carcasses either by direct deposition or indirectly through contaminated equipment, workers, installations, and air/water [10,53]. Operations conducted during cattle slaughtering, including bleeding, evisceration, and dressing, are associated with a great possibility of microbial contaminants as sterile muscles become exposed to microbial pathogens that were present on the skin, digestive tracts (dung), and equipment and in an unhygienic environment as well as due to non-compliance with proper slaughter processes and the lack of personal hygiene [10,54,55]. Also, Jaja et al. [56] explained that processed meats (ham, bacon, salami) are more vulnerable to contamination owing to the handling procedures, which include slicing, cutting, and repackaging into small portions, pieces, or slices, creating excellent conditions for further contamination, growth, and survival of the pathogens. Similarly, Saud et al. [57] highlighted that the improper handling of meat and processing and storing meat products at ambient temperatures by small shop owners could provoke further microbial growth and contamination. 

Apparently, foods originating from animals might harbour diverse microorganisms and the food can serve as a milieu wherein interactions occur between the pathogenic and the non-pathogenic bacteria during which drug resistance genes can be transferred by means of horizontal gene transfer, culminating in the emergence of novel bacteria demonstrating resistance to an antibiotic or multiple antibiotics [58]. According to Saud et al. [57], the new forms of the bacteria are better endowed with factors and structures that will cause them to become pathogenic to farm workers, animal health workers, workers at meat stores, and ultimately consumers via direct or indirect contact with the contaminated meat, animal, and manure.

From Table 1, it can be seen that the rate of prevalence of a particular pathogen can vary within locations/regions of a particular country and from one country to another. The different prevalence rates indicate different levels of pathogen/bacterial contamination, which can be ascribed to the differences in the hygienic and sanitary operations in the abattoirs, the environment (surroundings) within which slaughtering is conducted, the quality of the water utilised in the processing of the meat, the sampling season, differences in sampling methods, handling at the retail shops, and sample size as well as the culture/identification techniques. Therefore, we recommend strict monitoring of food for safety and the enforcement of proper regulations in the food sector in a bid to avoid future outbreaks of foodborne diseases.

**Table 1:** Prevalence of bacterial pathogens in meat and its product across some developing countries in the world.

Organisms	Sources	Prevalence (%)	Countries	References
*Staphyloccocus aureus*	Raw meat, quick-frozen meat, ready-to-eat meat (1850 samples)	35	China	Wu et al. [16]
*Salmonella enterica* subsp. *enterica*	Retail pork, beef, mutton, dumplings, and smoked pork (807 samples)	19.7	China	Yang et al. [59]
*Salmonella* serovars *Enteritidis*, *Hadar*, *Heidelberg*, *Stanley*	Beef products (400 samples)	1.25	South Africa (KwaZulu-Natal Province)	Naidoo et al. [60]
*Salmonella typhimurium*	Broilers	46.4	South Africa (North West Province)	Olobatoke and Mulungeta [35]
*Salmonella enteritidis*	Polonies	30.9		
*Salmonella newport*	Smoked viennas	22.9		
	(180 samples)			
*Salmonella* spp.	Beef (448 samples)	12.5	Southern Ethiopia	Wabeto et al. [61]
*Salmonella* spp.	Whole carcasses, feed, water, hand rinses (352 samples)	31.25	Bangladesh	Mridha et al. [62]
*Salmonella* spp.	Thigh and breast meat (broiler chickens, 80 samples)	20	Bangladesh (Mymensingh City)	Julqarnain et al. [63]
*Staphylococcus aureus*		36.8		
*Escherichia coli*		43.2		
*Salmonella* spp.	Liver, intestinal content, spleen, gall bladder (832 samples)	36.54	Egypt	Shalaby et al. [53]
*Salmonella* spp.	Beef (136 samples)	1.5	Nigeria (Abuja)	Bawa et al. [64]
*Escherichia coli*	Ready-to-eat meats (96 samples)	42	Namibia (Windhoek)	Shiningenin et al. [65]
*Staphylococcus aureus*		52		
*Listeria monocytogenes*		15		
*Shigella* spp.		06		
*Enterobacteriaceace*		83		
*Staphylococcus* sp.	African sausages (100 samples)	50.4	Kenya	Karoki et al. [66]
*Bacillus* sp.		19.5		
*Streptococcus* sp.		9.8		
*Proteus* sp.		2.4		
*Escherichia coli*		1.6		
*Coliform bacteria*	Raw chicken (200 samples)	97	Kenya (Nairobi)	Odwar et al. [67]
*Escherichia coli*		78		
*Salmonella* spp. *Staphylococcus* a*ureus*	Chicken meat and pork	42.1 29.1 but 14.7 contained both bacteria	Cambodia	Rortana et al. [68]
*Pseudomonas aeruginosa*	Raw, frozen, and imported meat (370 samples)	7.83	Iran (Alboz Province)	Rezaloo et al. [69]
*Staphylococcus aureus*	Chicken meat (1707 samples)	6.3	Thailand	Klaharn et al. [70]
Coliforms		13.5		
*Enterococcus*		24.7		
*Escherichia coli*		33.3		
*Salmonella*		33.4		
Coliforms	Chicken, pork, buffalo, and goat meat (50 samples)	84	Nepal	Bantawa et al. [18]
*Staphylococcus aureus*		68		
*Salmonella* spp.		34		
*Shigella* spp.		06		
*Vibrio* spp.		03		
*Pseudomonas aeruginosa*		40		
*Escherichia coli*	Chicken, goat, and buffalo (118 samples)	62.8	Nepal	Kumar et al. [71]
*Enterococcus* sp.		14.1		
*Klebsiella pneumoniae*		11.5		
*Escherichia coli* *Staphylococcus aureus*	Raw beef (40 samples)	32.5 20	Indonesia	Soepranianondo et al. [72]
*Staphylococcus aureus*	Chicken meat (60 samples)	58.3	Indonesia	Wardhana et al. [67] Wang et al. 2020 [73]
*Salmonella* spp.		48.3		
*Escherichia coli*		40		
*Staphylococcus aureus*	Beef (54 samples)	16.67	Ghana	Adzitey et al. [74]
*Escherichia coli*	Poultry (384 samples)	55.2	Tanzania (Dar es Salam)	Mgaya et al. [75]
*Salmonella*	Beef (117 samples)	21.4	Rwanda (Kigali)	Niyonzima et al. [76]
*Salmonella*	Goat/mutton, chicken, pork, and rabbit	19.6	Rwanda	Niyonzima et al. [77]
*Escherichia coli*	Goat carcasses (154 samples) from two slaughter slabs at Chinsapo-2 and Chigwirizano	29 and 38	Malawi (Lilongwe)	Tanganyika et al. [78]
*Bacillus* sp.		18 and 23		
*Proteus* sp.		15 and 13		
*Klebsiella* sp.		13 and 5		
*Escherichia coli*	Chicken meat, raw minced meat, and raw sausages (90 samples)	38	Zimbabwe (Harare)	Claudious et al. [79]
*Staphylococcus aureus*		19		
*Klebsiella pneumoniae*		08		
*Proteus mirabilis*		03		
*Citrobacter* sp.		01		
*Enterobacter* spp.		01		
*Enterococcus faecalis*		10		
*Coagulase* negative *Staphylococcus*		01		
*Salmonella* spp.	Crocodile meat (2749 samples)	0.5	Zimbabwe	Nhidza et al. [80]
*Escherichia coli* 0157	Meat cube samples	5.22	Botswana (Gaborone)	Magwira et al. [81]
	Minced meat	3.76		
	Fresh sausage (400 samples)	2.26		
*Campylobacter coli*	Imported and local chicken thighs (256 samples)	32.5	Benin	Kouglenou et al. [82]
*Campylobacter jejuni* *Campylobacter coli*	Chicken and turkey meat (85 samples)	68.531.5	Baghdad	Kanaan et al. [83]
*Staphylococcus aureus*	Cattle, sheep, and pig (237)	5.06	Lesotho	Seeiso and McCrindle [84]
*Salmonella* spp.		0.84		
*Escherichia coli*		6.33

(i) Assessing the microbiological safety of meat and its products

The consumption of food contaminated with microorganisms is still considered as the crucial pathway for foodborne infections in developing countries and these infections can be categorised into wide range of illnesses that are caused by viruses, parasites, or bacteria, as well as chemical contaminants [85]. However, animal and plant infections have been greatly implicated in the major economic losses that occur at the global level in the agricultural and food value chain industries and biodiversity. Therefore, the recognition of environmental, animal, and plant pathogens has become crucial in a bid to lessen and avert the transmission of diseases and expedite valuable management practices [15].

Notwithstanding, in order to ensure food safety, the key factor should be producing pathogen-free live animals, therefore enabling the slaughterhouse to keep the processing lines devoid of those microbes. However, monitoring of the microbiological level of raw meat appears to be a vital facet in sanitary management. Nevertheless, regulations and guidelines are compiled depending on the country to ensure that consumers are provided with safe meat and meat products [86].

Seeking to improve on the microbiological safety of meat and its products, the regulatory authorities have made it mandatory to employ “hazard analysis and critical control points” (HACCP) systems, which should be based on subjective evaluation of microbiological data that permit the numbers of indicator organisms to be estimated on the meat products at various stages of processing [87]. Evaluating the microbial contamination of meat carcasses at slaughterhouses and meat-processing points involves collecting samples either by excising or swabbing [88]. The quality and safety of the meat can be assessed while employing indicator microorganisms, and subsequently, aerobic plate counts (APCs)/total viable counts (TVCs)/standard plate counts (SPCs), coliform counts, and *Escherichia coli* counts can be measured [89]. Total viable counts give an indication of the overall viable/growing bacterial population estimated on a meat sample and express the microbiological quality of the meat, therefore, a higher TVC insinuates a poorer quality and shortened shelf life. In addition, coliform counts (CCs) and *E*. *coli* counts (ECCs) serve as indicators of faecal contamination of the meat and poor sanitation during processing. Generally, a higher level of these indicators (CC and ECC) correlate with a higher level of foodborne pathogens derived from faeces [89].

Microbial contamination of meat might result in intoxication (e.g., *Clostridium*, *Staphylococcus*) or infection (e.g., *Salmonella*). Moreover, the presence of some bacteria in meat can cause spoilage which can be manifested as off odours, off flavours, discoloration, and gas production (*Pseudomonas aeruginosa*). Sanlibaba [10] opined that to guarantee food safety, virulence traits causing food-poisoning outbreaks should be detected.

(ii) Isolating and identifying the specific bacterial pathogens

Table 1 shows the varieties of bacterial pathogen that most probably occur in different meat sources. Overall, bacterial pathogens in meat can be detected via culture-based and molecular methods, but enrichment of the meat samples is always conducted in broth upon arrival at the laboratory, after the meat is collected in a sterile plastic bag and transported while maintaining a cold chain [18].

In South Africa, as a developing country, cultivation on microbiological media is the most widely employed method in the laboratories, however, it is faced with many challenges as only viable and culturable bacteria will grow and reproduce on media, whereas spore formers and viable but non-culturable bacteria will be missed, therefore not giving the true richness of the microbial population or diversity of the microorganisms occurring in the meat samples [90]. This situation is unavoidable with culture-based methods as some organisms live in conditions that cannot be reproduced in the laboratory’s conditions owing to varied reasons as explained by other authors [91]. Notwithstanding, via the cultivation on microbiological media, whether enriched or selective or general-purpose media, microorganisms can be qualitatively and quantitatively detected. In the qualitative detection, the meat samples are enriched in different broth media depending on the bacteria of interest, employing brain–heart infusion broth, tryptic soy broth, buffered peptone water, Bolton broth, etc. Subsequently, a loopful of the culture of bacteria is streak plated on solidified agar plates and incubated at specified temperatures and for a specified number of days for the presumptive growth of the pathogens [59]. Similarly, for the bacterium to be detected, a certain portion of the meat sampled is introduced into the broth and homogenised; subsequently, about 0.1 mL can be transferred onto different media and incubated under specified laboratory conditions for the growth of bacteria, which are then identified either via biochemical methods or molecular methods, guided by the principles outlined in Bergey’s Manual of Determinative Bacteriology [92]. On the other hand, in quantitative detection, the bacteria are enumerated in the samples via total viable counts and most probable number techniques, after being introduced into broth and homogenised. In detail, a specific volume of the broth sample is diluted via ten-fold serial dilutions to obtain a series of decreasing concentrations, diluting the concentration of the viable bacterium in a bid to enable distinct colonial growth upon inoculation (spread plate method) on microbiological media and incubation at specified temperatures and for a specified number of days [18]. 

Alternatively, a metagenomics approach can be embraced as a tool to reveal the diversity of microorganisms in meat samples. According to Jagadeesan et al. [93], a thorough collection of genomes and genes recovered from microbes serving as a fundamental step in the accurate characterisation of the taxonomic and functional repertoire of microorganisms in meat as an environment will be obtainable via metagenomics. The authors revealed that the recovery of microbial genomes and genes via a metagenomics approach enables the characterisation of unknown microbiota in addition to offering opportunities for the prediction of the presence of pathogens in samples, resulting in a sustainable healthy food system. In addition, broad-range DNA amplification and sequencing of specific target sites of the 16S rRNA gene, with specific primers via polymerase chain reaction (PCR), can lead to the isolation and identification of both culturable and non-culturable bacteria [15]. However, this method is also disadvantageous in that PCR will amplify the DNA of every bacterial cell whether dead or living, be it relevant or not even a pathogen or a contaminant emerging from the meat or PCR reagents [91]. In conclusion, the above methods give an indication of the microbiological quality of the meat.

(iii) Bacterial pathogens identified in meat and its products

Philips et al. [94] noted that cattle are the primary reservoir for *Enterobacteriaceae.* Clearly, *E*. *coli* lives in the alimentary canal of animals and livestock are regarded as a first reservoir of the different species/strains of the organism with the same pathogenicity on the specified host [55]. The bacterium also has great potential in acquiring antimicrobial resistance. Consequently, the bacterium is considered as a sentinel in the surveillance programmes on antimicrobial resistance worldwide. Nevertheless, a high prevalence of intestinal commensals including *E*. *coli*, *Salmonella* spp., *Shigella*, and *Vibrio* sp. calls for concern from the public health sector, as the findings can be strongly associated with contamination stemming from poor hygiene and sanitation via the different stages of production until the meat product reaches the consumers by purchasing from meat shops and meat retailers [58]. 

In South Africa, Madoroba and colleagues [15] demonstrated a diverse and highly variable microbial community of different bacterial pathogens, including *Y. enterocolitica*, *Salmonella* spp., *L. monocytogenes*, *Campylobacter* species (*C. jejuni, C. coli,* and *C. lari*), *S. aureus*, *C. perfringens*, and *B. cereus*, in products originating from animals, including cattle, sheep, poultry, caprine, and game meat. More elaborately, a high prevalence of enteric pathogens in meat suggests unhygienic processing and inadequate sanitation conditions of meat shops. It has been demonstrated that direct contact with raw meat might create health risks to humans, especially slaughterers, emphasising transmission via the faecal–oral route [18]. Prominent bacterial pathogens causing serious threats to the food industry and that are well-known foodborne pathogens affecting the environment and public health include:

(a) Bacteria in poultry farming
  ***(i)***  ***Campylobacter jejuni*:** It is a Gram-negative, non-spore forming, fastidious, S-shaped or curved rod, belonging to the genus *Campylobacter* and classified under the phylum *Proteobacteria*, class *Epsilonproteobacteria*, family *Campylobacteriaceae*. *C*. *jejuni* and *C*. *coli* are the only known species of this genus implicated in human infections [95]. Other species do exist, making up 28 species, but are rarely involved in human infections*. C. jejuni* can persist in the environment and food, despite requiring minute atmospheric oxygen for growth. Likewise, the organism’s transition from the caeca of the birds to the environment causes perturbations due to exposure to atmospheric oxygen and temperature fluctuations. This could be explained by the fact that the bacterium is endowed with the potential to form biofilms and evolve into non-culturable but viable forms, facilitating its transmission from one human to another [96]. The organism has a unique defence system against oxidative stress by harbouring a copy each of alkyl hydroperoxide reductase (a*hpc*), catalase (*katA*), and superoxide dismutase (*sodB*). In addition, the peroxide stress regulator (*perR*) in *C*. *jejuni* directly regulates the transcription of the superoxide dismutase (SOD) enzyme*,* catalysing the breakdown of superoxide radicals, playing a key role as a defence against oxidative stress [97]. *C. jejuni* can equally evade immune responses owing to its ability to produce capsular polysaccharide (CPS), which contributes to its virulence. The ability of this organism to persist despite stresses indicates that the bacterium harbours complex virulence and fitness factors, which confer protection as well as aid the bacterium to sense, adapt, and compete in the changing microenvironment with sensors, signal molecules, adhesins for host receptors, and effectors for invasion and intracellular survival [98]. Like other bacterial infections, the production of proteinaceous virulence factors is required on the ribosome of the pathogen for the stages of adhesion to the host, survival in the host, and resistance to antimicrobials plus triggering disease [99,100].

The outer membrane of *C*. *jejuni* is composed of lipo-oligosaccharides (LOSs) that lack the O antigen that is common in most Gram-negative bacteria [101,102]. The presence of glycans (polysaccharides, CPS, LOS, S-layers) on the surface of the cells is crucial for host–cell interaction, enabling virulence and antigenicity [103]. The presence of phase-variable loci is very critical in this bacterium and they are located mainly in the CPS, LOS, and flagella, creating new structures that permit its evasion of the immune system and aid the organism to survive in varying environmental conditions [96,104]. The CPS is involved in invasion, adherence, intestinal colonisation, and systemic infection, resisting complement-mediated killing, whereas LOS is a mediator of invasion and adherence and activates Toll-like receptor 4-mediated innate immunity, resisting killing via cationic antimicrobial peptides [100]. The bacterium possesses a polar amphitrichous flagellum that is crucial to its pathogenesis by facilitating its movement, chemotaxis, adhesion, secretion of virulence factors, autoagglutination, and microcolony and biofilm formation plus evasion of the innate immune system [103]. In addition, the flagella promote avian colonisation and biofilm formation [99]. The organism displays great genetic heterogeneity, which causes strains to vary in adhesins, invasion routes, and invasion capacity (transcellular versus paracellular) necessary for the pathogenesis of the bacterium inside a host cell. Campylobacter adhesion to fibronectin (CadF) is a fibronectin-binding protein that demonstrates direct interaction with the extracellular matrix component fibronectin. CadF is a member of the family of microbial surface components recognising adhesive matrix molecule(s) (MSCRAMMs) that contributes to the pathogenesis of *C*. *jejuni* [105]. Other putative adhesins occurring in *C*. *jejuni* for adhesion include Campylobacter adhesion protein A (CapA) and JlpA protein [105]. Furthermore, it produces a cytolethal distending toxin (CDT) comprising three subunits (CdtA, CdtB, and CdtC) involved in halting processes occurring in the cell cycle [101]. 

Because of the organism’s ability to evade the immune responses together with its ability to survive in the host cells, in association with potential cellular components, this key zoonotic pathogen causes gastroenteritis in humans, termed as campylobacteriosis [106]. *Campylobacter* infection is reported to be most prevalent in the paediatric population (children aged under 5) with rates ranging from 2% in Sudan to 21% in South Africa [107]. Although *Campylobacter* infections are most common in children, other vulnerable populations, including the elderly and those with weakened immune systems, e.g., HIV/AIDS and cancer patients and transplant recipients, are also affected [108]. Igwaran and Okoh [109] noted a high incidence of *Campylobacter* species in raw meat procured from butcheries, open markets, and supermarkets in Eastern Cape Province of South Africa; thus, campylobacteriosis agents and the consumption of undercooked meat by consumers in this community are associated with health risks. Samuel et al. [110] emphasised that many developing countries, including South Africa, are hyperendemic with *Campylobacter* infections because of the inadequate food and environmental sanitation and the close contact between humans and animals in domestic settings occurring in rural and agricultural communities, amongst other factors. 

Poultry meat is regarded as the major source of human infections since the occurrence rate of *C*. *jejuni* is very high in avian species, owing to the high temperature of birds, an ideal condition for the growth of the bacterium. Due to the presence of the natural supply of nutrients and carbon sources in the lower intestinal tract niches, these sites appear as ideal niches for colonisation, supporting the robust growth and metabolism of *C*. *jejuni* [101]. Thus, chicken is a vital reservoir for the transmission of this organism as the organism could colonise the caeca of the chickens in enormously huge numbers [104]. According to Hakeem and co-authors [96], the intestinal mucus of avian species is sulphated and sialylated to a greater degree than that of humans, thus *C*. *jejuni* survives and reproduces in this site, wherein its pathogenicity is modulated to that of near-commensal bacteria in poultry. This explains the high load of *C*. *jejuni* existing in the guts of birds colonised after 2–3 weeks while they remain asymptomatic. This may suggest the high prevalence of the organism in commercial farms because of its shedding in faeces with subsequent faecal ingestion by other birds, emphasising bird-to-bird transmission as the main source of horizonal transmission on broiler farms [111]. 

The high recovery rate of this strain from the contamination of meat by the gut content is unavoidable due to the high number of this bacterium in the gut and the large population of the birds infected [112]. Notwithstanding, the prevalence of *Campylobacter* in poultry in addition to the contamination level of poultry products varies greatly from one country to another, explaining the need for different intervention strategies [113]. Its significance as an economic burden is not linked only to campylobacteriosis [114] but its long-term implication in the aetiology of Guillain–Barré syndrome, reactive arthritis, or post-infective irritable bowel syndrome. Most cases of campylobacteriosis are sporadic and self-limiting, hence, antimicrobial treatment becomes unnecessary. However, when the case needs hospitalisation, indicating potentially severe disease, antibiotics, including ciprofloxacin, azithromycin, erythromycin, and tetracyclines, are administered [115,116]. Nevertheless, cephalosporins should be avoided due to high resistance rates [103]. The ability of this bacterial species to display resistance to agents with antimicrobial activity indicates that it is a danger to the health of people [114]. In this light, Hakeem and colleagues [96] opined that strict biosecurity measures and vaccination, supplementation with probiotics, prebiotics, synbiotics, organic acids, bacteriocins, and quorum sensing inhibitors can improve the health condition of the guts of broilers and, via competition, exclude and reduce *C*. *jejuni* levels in broilers. Similarly, Steffan and co-authors [117] mentioned that bacteriophages are encouraging in the aspect of reducing *C*. *jejuni* in food production plants, describing phages as natural predators of bacteria.

***(ii)***  ***Staphylococcus aureus*:** This bacterium can be described as a Gram-positive, facultative, non-spore-forming, novobiocin-sensitive bacterium, which occurs as cocci, belonging to the genus *Staphylococcus* (comprising 36 species), family *Staphylococcaceae*, and order *Bacillales*. The organism, together with *Enterococcus* spp., *Klebsiella* spp., *Acinetobacter baumannii*, *Pseudomonas aeruginosa*, and *Enterobacter* spp., belongs to the group known as ESKAPE (an acronym used to describe six opportunistic, life-threatening nosocomial pathogens (*Enterococcus* spp., *S*. *aureus*, *Klebsiella* spp., *Acinetobacter baumannii*, *Pseudomonas aeruginosa*, and *Enterobacter* spp.) demonstrating growing multidrug resistance and virulence [118]. It is known to demonstrate the highest tolerance to reduced water activity and high salt concentration, enabling its survival in highly salted foods [119]. The authors proposed that changes in morphology, biofilm formation, transcriptome, metabolome, and virulence are responsible for the organism’s response and survival, establishing its colonisation. Therefore, it is found in a wide range of habitats, including the skin and nose, as well as on surfaces, indicating its distribution as ubiquitous in nature [120]. It is therefore a serious threat to hospital and community settings alongside the food industry [120]. Humans are the major reservoir of this pathogen.

The quick adaptability of this organism to environmental changes is one of the reasons for its high pathogenicity [119]. According to Velasco and colleagues [120], the bacterium possesses a plethora of virulence factors facilitating its colonisation of different sites in the human host, causing no infection. Its ability to cause several infections rests upon its capacity to evade the immunological responses triggered by the body’s system owing to invasion and colonisation. Notwithstanding, the organism produces an arsenal of virulence factors, including toxins, proteases, adhesins, and immune evasion factors [121]. The different virulence factors include extracellular proteins (cytolytic toxins (Panton–Valentine leucocidin, PVL, and haemolysins); enterotoxins (staphylococcal enterotoxins); SEA, SEB, SECn, SED, SEE, SEG, SEH, and SEI plus toxic shock syndrome toxin (TSST-1); exfoliative toxins (ETA and ETB), extracellular adherence protein (Eap), phenol-soluble modulins, microbial surface components recognising adhesive matrix molecules (MSCRAMMs), proteins (protein A and fibronectin-binding proteins—biofilm formation), teichoic acids, capsule, peptidoglycan, and enzymes (coagulase, staphylokinase, hyaluronidase, lipases, phospholipases, proteases, deoxyribonucleases), causing several infections presenting with different clinical manifestations when found in the bloodstream and internal tissues [122,123,124]. The clinical manifestations include infective endocarditis, bacteraemia, skin and soft infections, meningitis, gastroenteritis, toxic shock syndrome, septic arthritis, osteomyelitis, pulmonary infections, and a host of others, which are determined by the type of infection, strain type, and the site of infection [125]. The organism produces factors that disrupt the adaptive B cell and T cell responses and lessen protective immunity, therefore causing the infection to be recurrent throughout life [126]. 

The bacterium is considered as one of the major foodborne pathogens in fresh and ready-to-eat meat [16]. The different meat types studied in African countries include raw unprocessed beef, pork, goat meat, camel meat, lamb/sheep meat, showing varying levels of prevalence [118]. In South Africa, Blignaut et al. [127] noted different prevalence rates of 3.95% and 1.71% of *S. aureus* recovered from total mixed ration-based and pasture-based dairies. Similarly, Sineke and colleagues [128] registered a 68.8% prevalence of *S*. *aureus* in intensive pig production employing a farm-to-fork approach. On the other hand, Sigudu and co-authors [129] noted a variety of *Staphylococcus* species in the human population in the country, constituting 74.7% and 18.9% prevalence rates of coagulase-positive and coagulase-negative species, respectively, with males contributing 51.2% including mostly *S*. *aureus.* The treatment of *S. aureus* is marked by a history of development of resistance to antibiotics, including penicillins, methicillin, sulfonamides, tetracyclines, and glycopeptides (vancomycin) [130]. Methicillin resistance in the bacterium is very remarkable and the resistance to this drug occurs via the acquisition of the *mec* gene found on the staphylococcal cassette chromosome mec (SCCmec) element [131,132], causing methicillin-resistant *S*. *aureus* (MRSA). In a cross-sectional study conducted in South Africa, Govender and co-authors [133] reported a prevalence of 21% of MRSA amongst the *S*. *aureus* isolates recovered from various poultry meat products sold at abattoirs, meat-processing facilities, retail points, and cold stores at the main ports of entry into the country. Infections with MRSA show an increased trend in Sub-Saharan Africa whilst a slight decrease was observed in South Africa, Canada, the US, and Europe [134]. Methicillin resistance in this bacterium confers resistance to all beta-lactam antibiotics apart from the fifth-generation cephalosporins. Therefore, MRSA strain types are usually managed with drugs in other antimicrobial classes, but, depending on the country, vancomycin and teicoplanin are the antibiotics of choice [130]. Notwithstanding, vancomycin is still the last-resort antibiotic of choice for the treatment of MRSA [121].

***Salmonella* and *Yersinia* species:** These are Gram-negative, facultative, rod-shaped anaerobes, belonging to the phylum *Pseudomonadata*, family *Enterobacteriaceae*, and order *Enterobacterales*. They are both termed as enteropathogens causing diarrhoeal infections in humans and are grouped as zoonotic pathogens able to cause both water- and foodborne infections, negatively impacting the global community regarding public health, medical, and veterinary aspects [135]. Janda and Abbott [135] emphasised their recognised roles in gene structure and function, molecular and cell biology, and microbial pathogenicity. *Enterobacteriaceae* are noted for their acquired resistance to carbapenem antibiotics via enzyme synthesis, efflux pumps, and porin mutation [136]. The authors further mentioned that treatment regimens for bacterial infections are no longer successful, and infections are more problematic to manage or treat because of the existence of this group, the carbapenem-resistant bacteria.

***(iii)***  ***Salmonella* species:***Salmonella enterica* (*S*. *enterica*) and *Salmonella bongori* (*S*. *bongori*) are the two main species within a list of serotypes grouped into typhoidal and non-typhoidal serotypes based on the somatic O (lipopolysaccharides), surface Vi, and flagellar H antigens according to Kauffmann–White schemes [137]. The variability in the genetic characteristics of the pathogen ensures its survival and its spread in the environment and host [132]. Owing to the virulence of S. *enterica* subspecies *enterica*, this subspecies appears to be of significance, causing great distress [138]. The most common serotypes of *Salmonella* that can infect a broad range of hosts, including humans and birds, are *S*. *enterica* serovar *Typhimurium* and *S*. *enterica* serovar *Enteritidis*. Typically, *Salmonella* lives in the intestinal tracts of animals and humans and is released with faeces; however, larger quantities of *Salmonella* are shed from clinically sick animals than healthy ones, posing a great risk to humans [139]. The shedding of *Salmonella* together with faeces from food-producing animals is the principal route of contamination of the environment, feed, and water whilst the bacterial load in the intestines is the main mechanism of contamination of the animal carcass during slaughter [140]. The principal reservoir for non-typhoidal *Salmonella* is animals; moreover, Magwedere and colleagues [140] in a farm-to-fork food pathway in South Africa demonstrated that environmental samples (e.g., poultry houses, abattoirs, feed mills, water) were the main sources of non-typhoidal *Salmonella*. The isolates can survive in the environment for weeks or months owing to their tendency to form biofilms on contact surfaces [141], which protects against external physical (mechanical stress) and chemical treatment (antibiotics, disinfectants, etc.).

While the typhoidal serotypes (typhoidal salmonellosis) can invade the bloodstream, causing typhoid fever, in both animals and humans (e.g., *S*. *typhi* and *S*. *paratyphi*), the non-typhoidal serotypes are able to invade only the gastrointestinal tract, causing non-typhoidal salmonellosis (bacterial enteric illness/food poisoning), and can be transferred via animal-to-human and human-to-human routes (e.g., *S*. *typhimurium* and DT 104). Non-typhoidal *Salmonella* infections occurring in humans are considered as infections caused by *S*. *enterica* serovars different from *typhi* and *paratyphi* [140]. Nevertheless, typhoidal serotypes can be transferred via only the human-to-human route. There are disparities in the pathogenicity and hosts amongst the serotypes, therefore understanding the dominant serotypes, virulence factors, and genetic characteristics of the predominant strains in a setting (farm) can help in developing control measures [142]. Notwithstanding, every isolate is considered as a potential pathogen to all species because of the great possibility of isolating *Salmonella* from the environmental sources, food, animals, humans, and apparently healthy carriers [143].

Ensuing ingestion by humans, a portion of the bacteria demonstrate resistance to the low gastric pH, invading the intestinal cells. By forming *Salmonella*-containing vacuoles (SCVs) internalised by macrophages, the bacterium evades destruction by the immunological response and survives and grows intracellularly [144]. The SCV is a central feature in the bacterium’s survival. In addition, the organism has the ability to employ alternate sources for the provision of nutrients needed for its survival, e.g., glyoxylate cycle, operated by two unique enzymes, malate synthase and isocitrate lyase. The glyoxylate cycle is paramount in survival during oxidative stress and pathogenesis of the microorganism [145]. Osmoregulation, dormancy, cross-protection, and cross-tolerance are the means through which *Salmonella* species survive in foods of low water activity [146]. Also, the multifactorial and complex virulence determinants in *Salmonella* support its survival in the host. More than 200 virulence factors facilitate attachment, invasion, macrophage survival, multiplication, and, lastly, systemic dissemination, constituting the five stages of pathogenesis caused by *Salmonella* [147]. These include flagella, fimbriae, cellulose, and effector proteins. Furthermore, host invasion and intracellular survival of the pathogen are based on T3SS1 and T3SS2, type III secretory systems encoded by *Salmonella* pathogenicity islands SPI-1 and SPI-2, translocating separate sets of effectors proteins [144,146]. Forty (40) core effectors are produced by SPI-1 and SPI-2, but with the majority from SPI-2 (*Avr*A, *Gog*A, *Gog*B, *Gtg*A, *Gtg*E, *Pip*A, *Pip*B, *Pip*B2, *Sif*A, *Sif*B, *Sip*A, *Sip*B, *Sip*C, *Sip*D, *Slr*P, *Sop* A, etc.), displaying critical virulence in different hosts [142]. The invasion gene (*inv* A) found on the pathogenicity island of the bacterium serves as a biomarker in detecting the bacterium and promotes virulence as it participates in the invasion of the epithelial cells in the host. The bacterium produces an enterotoxin (*stn*) that equally serves as a biomarker differentiating between *S*. *enterica* strains, *S*. *bongori*, and other members of the family *Enterobacteriaceae*. Fimbriae encoded by the gene *fim*A are filamentous structures on the surface of the bacterium engaging in the invasion of host epithelial cells [148]. The organism carries virulence plasmids, collectively denoted as *pSV* plasmids, relevant in virulence. *Salmonella* plasmid virulence (*spv*) harbours the *spv*R gene and presents as the signature locus on *pSV* [149]. An outbreak of foodborne illness took place in Mpumalanga Province, South Africa, indicating the occurrence of non-typhoidal *Salmonella* (NTS) serotypes consumed through meals produced from poultry [150]. These outbreaks may present as threats to public health [36], as NTS usually manifests in children and adults, particularly in sub-Saharan countries, including South Africa, as gastroenteritis (inflammation of the GI tract) and/or as infections of the bloodstream.

Through retrospective laboratory-based surveillance conducted in South Africa, Gelaw and colleagues [140] noted 1229 *Salmonella* isolations from non-food and food animals, with 83.5% total isolations occurring in food animals, including 72.7% of cattle and poultry and 1.3% of pigs. Similarly, Ramhatal et al. [39] recovered via genomic sequencing a rare serotype, *S*. *Yoruba*, alongside *S*. *Heidelberg* and *S*. *Kentucky* amongst the farm and abattoir samples in their investigation from the farm-to-fork continuum of an intensive poultry farm in the same province. On the other hand, Gallichan et al. [151] registered a 58.33% prevalence of *Salmonella enteritidis* clades causing invasive disease in the human population of South Africa, attributing this finding to the rising consumption of poultry and importation to the country. However, in animals, the occurrence of *Salmonella* infections is self-limiting and affected by the age of the animals, husbandry, and management-linked factors in intensive farming [152]. In South Africa, the control measures undertaken to regulate the occurrence of non-typhoidal *Salmonella* in food-producing animals are aimed at *Salmonella enterica* subspecies *Enterica* serotype *Enteritidis* in poultry in Section 31 of the Animal Diseases Act (Act 35 of 1984) [140]. The treatment of choice for *Salmonella* infection upon diagnosis and antibiotic susceptibility testing is fluoroquinolones or azithromycin.

(b) Bacteria in Livestock farming
  ***(iv)***  ***Yersinia enterocolitica:*** This bacterium, *Y*. *pestis*, and *Y*. *pseudotuberculosis* are the three (3) main pathogens causing human diseases which are found amongst the sixteen (16) species grouped under the genus *Yersinia*. The key features of this group include, firstly, that the species are able to grow and survive at refrigeration temperatures, with great implications for contamination of food stored at such temperatures, which will ultimately affect the health of humans. Therefore, they are described as life-threatening bacteria that can cause foodborne infections [153]. Secondly, infection with *Yersinia* sp. is termed yersiniosis (zoonotic infection of the intestines) and the individual continues with shedding of the pathogen in his/her faeces for 3 months even when the individual no longer presents symptoms. Thirdly, members of this group do not demonstrate the ability to chelate iron, however, they employ siderophores produced by other organisms so as to chelate iron, an essential growth factor [154].

*Y*. *enterocolitica* is a non-spore-forming, urease-producing coccobacillus, motile with a peritrichous flagellum, that can be broken down into six (6) discrete groups, including 1A, 1B, 2, 3, 4, 5 biotypes and 70 serotypes in relation to pathogenicity and geographical distribution; the biotypes are further demarcated into three (3) groups based on the measure of pathogenicity, into non-pathogenic (1A), moderately pathogenic (2–5), and highly pathogenic (1B) [155,156]. *Y*. *enterocolitica* subsp. *enterocolitica* (also known as the American bioserotype) and *Y*. *enterocolitica* subsp. *Paleartica* (also known as the European bioserotype) are the two subspecies of this species based on 16S rRNA and genomic studies [153]. 

Like *Listeria*, the bacterium is psychrotrophic and has the potential to survive outside the host environment. The ability to grow within or outside the human body is described as a biphasic lifestyle. The organism is ubiquitous in nature, distributed in food, water, and animals. It is transmitted via the faecal–oral route alongside contact with animals and contaminated food. The primary reservoir is pigs, with the pathogen isolated from tonsils, lymph nodes, tongues, intestines, and faeces, although they are asymptomatic [157]. The virulence determinants in *Y*. *enterocolitica* are both plasmidal and chromosomal. The most well-known and crucial virulence determinant in the bacterium is the plasmid of *Yersinia* virulence (pYV), whose presence in some strains causes their migration from Peyer’s patches to mesenteric lymph nodes and internal organs, where they multiply, leading to the formation of necrotic abscesses [155]. *Y*. *enterocolitica* isolates categorised as biotypes 1B and 2–5 have their pathogenicity ascribed to the presence of chromosomal and 70 kb pYV plasmid genes. In addition, the biotype 1B harbouring pYV carries a chromosomal high-pathogenicity island (HPI) connected with yersiniabactin (an iron acquisition system) that enables the uptake and consumption of iron, even in instances of limited iron availability [158]. The lipopolysaccharide of this bacterium, which is encoded chromosomally, can transform from smooth to rough and acts as an endotoxin following the rupture of the bacterial cells [155]. Biotype 1A is primarily non-pathogenic because of the lack of the virulence-associated factors of pYV [158]. Like other Gram-negative bacteria, *Y*. *enterocolitica* employs the type III secretory system (T3SS) in different ways to survive in the varying environments and to enhance pathogenicity inside the host [159]. Pha [160] emphasised that the bacterium utilises its T3SS to thwart phagocytosis, promote distribution, as well as inhibit responses due to inflammation. The T3SS protein complex, termed as the injectisome, translocates effector proteins from the bacterium to the host cells [159]. *Yersinia* infection occurs after ingesting contaminated plant- and animal-based products, manifesting with symptoms ranging from as a mild but self-limiting gastroenteritis to acute mesenteric lymphadenitis, appendicitis, diarrhoea, ileitis, and septicaemia.

However, the incidence of yersinosis is low due to the high infection dose involved as well as the lack of selective diagnostic methods. Likewise, Shoaib and colleagues [161] reiterated that the growth habits, the low level of the pathogen in samples, its similarities to other pathogens in terms of morphology, as well as the lack of rapid, cheap, and accurate detection approaches mean *Y*. *enterocolitica* remains a challenge to food handlers and researchers. In South Africa, Robin-Browne et al. [162] noted 14 patients with *Y*. *enterocolitica* infections over a year (between 1966 and 1967). However, in the country, there is a paucity of knowledge of the prevalence of the organism in retail meat and its products, although Madoroba and colleagues [15], in a recent investigation, registered a prevalence of 17% amongst samples comprising meat and meat products. Subsequently, in a qualitative study, Seakamela and co-authors [163] noted a 12% prevalence of *Y*. *enterocolitica* isolates classified under biotype 1A, with the majority harbouring virulence genes, including *ymo*A (*Yersinia* modulator), *yst*B (encodes *Yersinia* heat-stable enterotoxin), *fep*D (enterochelin receptor protein), ail (attachment invasin locus), *fep*A (enterochelin receptor protein), *inv*A (invasin), and *myf*A (encodes a fimbrial and putative adhesin) among meat and meat products procured across retail outlets in South Africa. The authors further mentioned the predominance of *bla*_TEM_ and *cml*A with high resistance to amoxicillin, ampicillin, and cephalothin. 

Treatment of yersiniosis, depending on the individual (age), entails antibiotics belonging to the classes aminoglycosides and trimethoprim–sulfamethoxazole [154].

***(v)***  ***Listeria monocytogenes***: It is a bacterial species amongst twenty other (20) members of the genus *Listeria*. This bacterium and *L*. *ivanoii* are grouped together, infecting humans and animals, however, the latter is an animal pathogen. *L*. *monocytogenes* is classified as a Gram-positive, rod-shaped, facultative anaerobic bacterium belonging to the phylum *Firmicute*, order *Bacillales*, class *Bacilli*, and family *Listeriaceae* [164]. A significant level of diversity is said to occur within *L*. *monocytogenes* as the organism has evolved slowly from four major evolutionary lineages denoted as I to IV and represented by 14 lineage-related serotypes and over 170 clonal complexes as defined by multilocus sequence typing and whole genome phylogenetic analysis [165]. Malakar and co-authors [166] explained that the strains belonging to lineage II (serotypes 1/2b, 3b, 4b, 4d, 4e, and 7) are usually isolated from food and animals with listeriosis while the strains associated with lineage I (1/2a, ½c, 3a, 3c, and 4h) are responsible for the outbreak of listeriosis in humans. In addition, the stains grouped under lineages III (4a, atypical 4b and 4c) and IV (4a and 4c) are typically recovered from animal sources but are somewhat rare.

Strain divergence of high magnitude based on the potential of virulence, adaptation to the environment, and responses to stress occurs in the bacterium. Multiple clonal complexes occur in *L*. *monocytogenes* with highly diverse, epidemic potential [167]. The complex adaptability amongst the different strains of the bacterium can be ascribed to the genes and genomic islands responsible for virulence and resistance to environmental stress [168]. The clonal complexes, viz., CC1, CC2, CC4, and CC6, are hypervirulent in humans and they belong to lineage I; thus, the lineage I strains are responsible for the majority of the outbreaks. On the other hand, the clonal complex CC7 is of medium virulence and associated with lineage II [169] whilst CC9 and CC121 are less virulent. Quereda et al. [170] highlighted that *L*. *monocytogenes* has the capacity to incite its internalisation by non-phagocytic cells, causing it to dodge the intestinal epithelium, the blood–brain barrier, and the placenta. These are very crucial pathophysiological barriers to surviving as well as replicating in phagocytes. The authors further noted that the cell wall and its metabolism are critical factors in the virulence of the bacterium. Most of the virulence effectors are proteins that are located on the surface of the bacterium in collaboration with the cell envelope or secreted to the extracellular milieu. 

Matle and colleagues [171] remarked that *L*. *monocytogenes* harbours a suite of virulence factors responsible for its pathogenicity and subsequent infection, however, most of these determinants are clustered along the chromosome in the genomic island or islands or *Listeria* pathogenicity island-1 (LIPI-1). Disson et al. [172] revealed that the *Listeria* pathogenicity island ranges from I to IV. The host of virulence factors identified in the organism include listeriolysin O(LLO), a surface actin assembly-inducing (*Act* A) protein, internalins (internalins A, B; InI A, InI B), and two distinct phospholipases (phosphatidylcholine-specific phospholipase (PC-PLC) and phosphatidylinositol-specific phospholipase C (PI-PLC)) [173]. *L*. *monocytogenes* produces extracellular vesicles implicated in toxin release, virulence, and transference to host cells, distributing antibiotic resistance and stimulating immune responses. Coelho and colleagues [173] reported that extracellular vesicles contained most of its virulence proteins. LLO is a cholesterol-dependent pore-forming haemolysin and it is regarded as the major virulence factor needed for the survival of the bacterium in the intracellular space as well as for inducing apoptosis in lymphocytes [174]. In addition, internalin B disrupts macrophage function in an idInlB isoform-dependent manner. Moreover, PC-PLC and PI-PLC, together with LLO, cause the disruption of the single vacuolar membrane, leading to the release of the bacterium into the cytoplasm of the host cell [173]. The organism employs flagella for uniform movement within its environment [175] as well as the invasion-associated protein (IAP) denoted as protein p60, which is a murine hydrolase enzyme essential in septum separation during the last stage of cell division [171].

The pathogen is ubiquitous in nature, occurring in a wide range of environmental samples, involving hostile conditions of the environment of low pH, high pH, low temperature, ultraviolet light, elevated concentration of salt, as well as the presence of biocides plus heavy metals [176]. This ability creates threats to the food industry and consumers because the organism has the potential to tolerate extreme environmental conditions by means of regulating the cytoplasmic fluidity and modify the composition of lipids, as well as genetic factors that confer the ability to form biofilms, facilitating its colonisation and persistence in food-processing industries [165]. Since it is widely distributed in several environments, the organism’s transmission into food-processing plants by means of employees, raw materials, and equipment establishes long-lasting colonisation owing to its capacity to survive in various stressful conditions (tolerating disinfectants and sanitisers) and to form biofilms [168]. The stress conditions are said to affect the virulence of the different strains differently [170]. It is amongst the deadliest foodborne pathogens, presenting as a prominent hazard to the food industry since it can survive in extremes of environmental conditions and physiological stresses due to its inherent adaptability, persistence, and, ultimately, ability to cause an infection in humans and animals called listeriosis. 

Listeriosis causes great mortality in the vulnerable population, including neonates, the elderly, children, pregnant women, and immunocompromised individuals [177]. The outbreak of listeriosis can be sporadic and epidemic and caused by consuming contaminated foods, including salads, vegetables, meat products, milk and other dairy products, and ready-to-eat foods [178]. South Africa has registered the most cases of listeriosis between January 2017 and July 2018, wherein 937 cases were identified affecting different fractions of the population, including HIV patients and pregnant women [179]. Furthermore, the authors, with the help of whole genome sequencing, traced the outbreak to a ready-to-eat processed food source (polony), concluding that in a middle-income country with a high prevalence rate of HIV infection, the organism is able to cause uneven illnesses among pregnant girls and women alongside HIV-positive individuals. According to Manyi-Loh et al. [180], a proper diagnosis precedes an appropriate treatment and the treatment in humans of *L*. *monocytogenes* entails antibiotics, including gentamicin, ampicillin/amoxicillin, chloramphenicol, penicillin, tetracycline, rifamycin or trimethoprim, and sulfamethoxazole employed either as a standalone or a combination therapy. Elsayed et al. [181] opined that based on the sample type, the strains can vary in their susceptibility to antibiotics. Similarly, Matle and colleagues [171] reported the presence of *L*. *monocytogenes* (14.7%) in meat sold in local markets and received at the three entry ports into South Africa, with 1.7% characterised with multidrug resistance against 13 to 19 antibiotics. 

It is worth concluding that food safety in terms of microorganisms has attracted increased public health attention globally and the implication of food as a vehicle of transmission of many diseases has been recognised for many decades, particularly in developing countries, wherein there are weak regulatory systems, inadequate food safety laws, and a lack of quality education for food handlers, and hygienic standards and sanitation have been greatly compromised [64]. Therefore, food safety is very significant for healthy living. 

#### 2.2.2. Chemical (Antibiotics) Contamination of Meat 

Livestock (pigs and cattle) farming and poultry farming are becoming more industrialised, incorporating the increasing use of enormous volumes of antibiotics in a bid to fulfil consumers’ increasing demand for chicken and beef. Intensification of food animal production systems is linked with huge antibiotic consumption. Saraiva et al. [182] noted that the amount of antibiotics consumed in livestock farming is anticipated to double in some countries, including Brazil, Russia, India, China, and South Africa (BRICS). The communal animal farming operated in South Africa embraces challenges, involving high disease burden and inadequate veterinary extension services. To address these, the government of the country through the Fertilizers, Farm Feeds, Agricultural Remedies and Stock Remedies Act 36 of 1947 endorse the purchase of some antibiotics including tetracyclines, sulfonamides, cloxacillin, intramammary fosfomycin, tylosin, and kitasamycin over the counter (OTC). About 29% of all the available antibiotics occur in the form of premixes and are frequently used to treat and prevent diseases in poultry and pigs as well as for growth promotion. This resolution is to enable timely management of easily recognisable endemic diseases [183]. This means livestock farmers have the leverage to access certain antimicrobials without supervision from any veterinarian [48]; this heightens the imprudent use of antimicrobials, which drives the development of antibiotic resistance. Moreover, the South African Veterinary Association published guidelines on the use of antimicrobials in pig farming performed in the country as well as encouraged the use of critically and highly important drugs that are crucial and relevant in human medicines, namely, streptomycin, gentamicin, ampicillin, erythromycin, ciprofloxacin, and tetracycline, for pigs. Surprisingly, Moyane et al. [184] noted that there is a scarcity of data on the volume of antibiotics employed in livestock farming in South Africa and data on the patterns of consumption of antibiotics are lacking. Accordingly, antibiotic usage in animal farming is primarily for growth promotion, employing them at subtherapeutic doses to improve feed conversion efficiency, the quality of the carcass, and economic production or for replacing very costly hygienic measures [156].

Chemical contamination can be viewed as contamination with antibiotic residues, which are chemical substances. Tadesse and Tadesse [185] defined residues as all the active metabolites that remain in the meat or food products originating from animals to which medications were administered. The quantity and the type of antibiotics administered to the animals play a significant role, including the mode of administration as different antibiotics with different chemical structures are metabolised differently. It is emphasised that amongst the modes of administration, including oral, parenteral, or topical, it is through injections that antibiotic residues exceeding thresholds are encountered because the antibiotics might accumulate in the adipose tissues, dodging the metabolism and the elimination of the drugs, therefore causing the drugs to persist in the tissues of the animals even after slaughter.

In addition, Getahun and colleagues [186] pointed out that antimicrobial residues might occur in meat and meat products via different practices, including the abuse of chemotherapeutic agents, violating withdrawal periods even with the proper administration of the anti-infective agent, alongside the use of antibiotics as growth promoters and feed additives. Darwish and colleagues [187] affirmed that antibiotic residues are recorded extensively in animal-derived foods in Africa, with levels exceeding WHO maximum residue levels, emphasising tetracyclines as the most predominant prescribed drug (41%), followed by beta-lactams at 18%. In 2017, Ramatla and colleagues [188] evaluated antibiotic residues in raw meat in Mafikeng, South Africa using ELISA, TLC, and HPLC; in relation to sulfonamide, tetracycline, streptomycin and ciprofloxacin, the concentrations ranged from 19.8–92.8, 26.6–489.1, 14.2–1280.8, and 42.6–355.6 µg/kg with ELISA, while HPLC detected ranges of 20.7–82.1, 41.8–320.8, 65.2–952.2, and 32.8–95.6 µg/kg, respectively. 

The occurrence of antibiotic residues in meat and its products have direct adverse effects on human health upon consumption in addition to the indirect consequence of antibiotic resistance [189]. Furthermore, van Boeckel and colleagues [190] highlighted that the trend in antimicrobial resistance is poorly documented in low- and middle-income countries. Ayukekbong and co-authors [191] mentioned that unsuitable prescription practices, insufficient patient education, limited diagnostic facilities, illegal sale of antimicrobials, the lack of appropriate functioning drug regulatory mechanisms, and the non-human use of antimicrobials (animal production) are some of the factors influencing antibiotic resistance. The way antimicrobials are used in the food animal industry in relation to the classes of antimicrobials, the doses administered, as well as the purpose will have a huge impact on the emergence and distribution of antimicrobial resistance [182]. Thus, the animals are serving as reservoirs of antibiotic-resistant microorganisms and encourage the proliferation and distribution of pathogenic microbes in addition to antibiotic resistance [57]. Notwithstanding, antibiotic-resistant bacteria have also been isolated from farms, particularly from animal manure, drinking water, feed, etc. [192]. Table 2 shows different bacteria and their percentages of multidrug resistance and antibiotic resistance genes recovered in studies conducted with samples from animal farming in South Africa.

Table 2 shows multidrug resistance demonstrated in bacterial pathogens found in meat, faeces, wastewater, and irrigation water in the different provinces of South Africa. There is persistent interaction between humans, food, animals, and the environment. South Africa is a water-scarce country and water recycling is one of the options to make water available for utilisation, e.g., water is used for irrigation of crops or grass utilised by animals as food, therefore if contaminated water is used, it will ultimately enter the food chain ending in the consumers [202]. The country is equally faced with a very high burden of infectious diseases, and a quadruple burden of disease, including HIV/AIDS, tuberculosis, diabetes, and hypertension and other organ-associated or non-communicable diseases [206]. Specifically, South Africa has a large HIV/AIDS population, who rely on the administration of antibiotic drugs to enhance their immunity. These individuals often experience recurrent gastroenteritis owing to infection with opportunistic bacteria. Regular administration of antibiotics to this population as treatment can result in the development of antibiotic resistance in the disease-causing bacteria because antibiotic resistance occurs, naturally, wherever antibiotics are in use [207]. The One Health approach includes human, animal, and environmental health, which are interconnected or inseparable; this means that the health of one of the three components affects the other two, therefore, the antibiotic resistance emerging in the clinical (human) setting can influence the others. In this light, antibiotic resistance can be transferred to animal and environmental (plant, food, water, soil) bacterial species via horizontal gene transfer.

The burden of AMR is disproportionately higher in low- and middle-income countries, although it affects every country. Table 2 shows findings from studies conducted in the different provinces and in specific locations/regions within each province. Nevertheless, discrepancies are displayed in the prevalence of antibiotic resistance in some zoonotic pathogens. These discrepancies cannot be associated solely with animal farming but are suggested to be attributed to the following:

Factors influencing the differences in percentage prevalence of antibiotic resistance between the provinces of South Africa:

- Overall, the antibiotic resistance profile or susceptibility profile of a bacterium varies with time, source of sample, strain type, climate change, and geographical location (different regions and different provinces in South Africa) [208]. This is because the bacterium develops antibiotic resistance over time caused by mutation in the DNA of the bacterium and the transfer of antibiotic resistance genes from one bacterium to the next via lateral or horizontal gene transfer [207]. The topography of South Africa ranges from desert to semi-desert to subhumid and wet, but half of it is arid or semi-arid. The country experiences both subtropical and temperate climatic conditions affected by the ocean and the interior plateau. It is highly vulnerable to changes and variations in climatic conditions because of the country’s reliance on rain-fed agriculture and natural resources [209]. The nine provinces of the country harbour different population sizes and experience the four climatic seasons (autumn, spring, winter, and summer) [210] but to different magnitudes as major differences in climate can be found from one region to another. This could be attributed to the geography of the region, anthropogenic activities, as well as the behaviour of the people [211]. This significantly affects the prevalence of antibiotic resistance in bacteria as an increase in temperature has a demonstrated effect on bacterial growth and its ability to transfer genetic material that encodes antibiotic resistance [212].
- Political and economic factors: The consumption patterns of antibiotics employed in agriculture vary across regions and countries within the developing world as their use is guided and regulated by the antibiotics policies of each country [213]. The governance and economy vary from one province to another [210]. Due to poor governance and economy, the lack of infrastructure pertaining to health care, sanitation, water, and hygiene has effects on antimicrobial resistance [214]. Adequate facilities to enforce sanitation and hygiene will cause a dramatic decrease in diarrhoeal diseases treated with antimicrobials. Antibiotic use appears as the main selective pressure encouraging the emergence of antibiotic resistance [207]. Achoki and colleagues [215] reported marked provincial health inequalities and explained that South Africa operates a federal system, wherein provincial governments decide their own health priorities, which in turn describe their health competency. Weak governance culminates in minimal attention directed to the status and functioning of the health system, poor regulations in antimicrobial stewardship, and a lack of monitoring of antimicrobial consumption and surveillance systems involved in antimicrobial resistance [216]. Moreover, the effective management of wastes (domestic, industrial, hospital, agricultural) varies with the different municipalities of the provinces and the state of wastewater treatment plants.
- Sociological factors: Impoverished education and awareness tend to lead the population to believe common myths, cultural practices, and belief systems [217]. Poverty, cultural, and social factors also influence the people’s consideration of self-medications against common infections, the purchase of medications over the counter or from unregulated drug dispensaries, visits to traditional practitioners, or even the borrowing of medicines from their neighbours [214]. Most of the South African population has embraced traditional health practitioners as a vital group in their health care [218]; however, this practice tends to vary across the provinces. According to Porkharel et al. [214], the traditional healers provide patients with medications prepared from unknown chemical agents mixed with suboptimal concentrations of conventional antibiotics, which equally provoke antimicrobial resistance.
- A farmer’s individual attitude, knowledge, and practice of the use of antibiotics influence animal farming and their waste management practice/systems [219]. Every individual is unique in his/her perception and behaviour. Moreover, the different provinces vary in their economic and social status [210] with Eastern Cape being considered the poorest amongst all the provinces of the country and the inhabitants often resort to agriculture and natural subsistence for their livelihood [40]. Although the government has given guidance and policies on the types of antibiotics to be implemented, human behaviour tends to vary, which will eventually affect antibiotic consumption, ways of managing the animal wastes containing excreted or residual antibiotics, antibiotic-resistant bacteria, and their resistance genes. In a study conducted in Mpumalanga, South Africa, Mupfunya and colleagues [48] witnessed knowledge gaps amongst farmers relating to prudent antimicrobial practices and antibiotic resistance that resulted in antibiotic resistance levels between 8 and 16% for *E*. *coli* and 3 and 55% for *Enterococcus* isolates.

In this light, multidrug resistance in foodborne bacteria is a crisis and equally affirms it is a global challenge that merits coordinated responses to mitigate further increases in antibiotic resistance. MDR poses a serious threat to humans, and it is a public health menace. To circumvent the spread of AMR, it is of utmost importance to have knowledge on the origin/emergence of AMR, i.e., to understand the sources of origin or ways of development of AMR [214]. Therefore, all potential sources of MDR bacteria should be noted and methodologies devised to lessen their occurrence in meat and its products. Of utmost importance is limiting the overuse or abuse of antibiotics in farm animals via repeated exposure of the animals to minute levels of antibiotics, which contributes to antimicrobial resistance. This is because some of these antibiotics are surrogates or the same as those employed in human medicines for treatment [220]. The level of multidrug resistance noted in South Africa in conjunction with the weak regulatory surveillance system is of great concern to humans, animals, and the environment. Therefore, a wide, local, and regional antimicrobial resistance observatory system is needed. This will also require collaborative strength to fashion local, regional, national, and global contingency plans to contain antimicrobial resistance as there are no geographical boundaries to prevent the distribution of antibiotic resistance via trade, travel, etc. Routine surveillance of antibiotic resistance in bacteria is a vital early warning of epidemiological significance, nevertheless, the development of antimicrobial resistance in pathogenic and zoonotic bacteria that exert effects on the health of people and animals further heightens the quest for more strengthened observation and monitoring [26].

##### Extensively Drug-Resistant (XDR) and Pan-Drug-Resistant (PDR) Bacteria

Resistance is an action demonstrated by microorganisms against antibiotics, wherein the antibiotic becomes ineffective in blocking one or several pathways that are involved in protein, folate, nucleic acid, or cell wall synthesis that are essential for the survival of the bacterium [221]. The drug resistance exhibited by microorganisms can be categorised into three groups, namely multidrug resistance (MDR) defined as non-susceptibility of a bacterium to three antibiotics or more in different antibiotic classes [222]. Extensive drug resistance (XDR) describes a microorganism displaying resistance to at least one agent in all classes but remaining susceptible only to one or two antibiotic classes, while pan-drug resistance (PDR) refers to the non-susceptibility of a bacterium to all the agents in all the antibiotic categories important to the treatment of a specific bacterial infection [223]. Several authors have demonstrated MDR, XDR, and PDR in *K*. *pneumoniae*, *E*. *coli*, *P. aeruginosa,* and *A. baumannii* [221,224]. Overall, Adrizain et al. [225] highlighted that the bacteria belonging to the ESKAPE group exhibit XDR and PDR. Owing to their strong viability/stability, incidence rate of colonisation, and resistance to a host of antimicrobials, XDR and PDR bacteria are reportedly isolated from hospital and environmental settings [226]. The menaces of drug resistance in these different categories seem to be huge or great, moving from MDR through XDR to PDR as the number of antibiotics to which the bacterium demonstrates resistance increases. Accordingly, XDR involves resistance to more antibiotics than MDR, and acquiring additional resistance mechanisms offers potential to develop to PDR. Consequently, the treatment options are limited in relation to the level of resistance, i.e., XDR and MDR, as the development of new antibiotics is not commensurate to the rate at which bacteria develop resistance, necessitating the use of relatively toxic drugs, high doses of the drugs, or drug combinations [227]. Karakonstantis and colleagues [224] emphasised that a synergistic drug combination is the only treatment option for pan-drug-resistant *A*. *bamannii.* In this light, infections with these drug-resistant bacteria often receive initial inappropriate antibiotic therapy, leading to worse outcomes and greater mortality [228]. It is explained that timely and appropriate antibiotic therapy, described as an antibiotic regimen with *in vitro* activity exerted against the disease-causing agent, is the very first and salient step in optimising the outcomes of patients with serious infection. Since PDR organisms easily adopt resistance mechanisms, the choice of treatment involves older and highly toxic agents, including polymyxin and tigecycline, leaving restricted and suboptimal options for treatment [226]. In current times, more MDR bacteria exist than XDR and PDR bacteria [222].

According to Dafale et al. [229], over 60% of human infectious diseases are caused by zoonotic bacteria. It is equally clear that huge quantities of antibiotics are employed in animal farming, resulting in the generation of drug-resistant zoonotic bacteria. Drug-resistant microorganisms tend to complicate treatment regimens of patients, causing clinical and financial burdens on health care providers and the patient [222]. Infection caused by an MDR bacterium often results in prolonged illness and hospitalisation as well as mortality. Accordingly, the costs of treatment and hospitalisation over long periods are more than double compared to cases without MDR. In relation to XDR and PDR, the costs will be highly amplified above those of MDR, therefore leading to greater socioeconomic burden. 

The level of resistance, i.e., XDR and PDR, heightens future concern due to the lingering increase in antimicrobial resistance, especially in the Gram-negative bacteria found in water and food, and a dearth of new agents in the developmental pipeline [228]. Through the One Health concept, it is apparent that antibiotic resistance originating in food will ultimately affect humans. Therefore, the medical communities are faced with threats from untreatable infections caused by XDR and PDR bacteria, including *Klebsiella pneumoniae*, *Pseudomonas aeruginosa*, and *Acinetobacter baumannii* [230]. Accordingly, Ozman et al. [226] purported that these types of drug resistance are responsible for several challenges in society and cause difficulties in treatment, encouraging the development of novel drugs by doctors. This can be explained by the fact that it is not easy to develop a standard therapeutic regimen to manage PDR infections in ill individuals [231].

## 3. Origin and Dissemination of Antimicrobial Resistance through Animal Farming to Humans

Treatment involving antibiotics is one of the predominant strategies existing in human medicine to combat bacterial infections. In animal farming, it is well documented that a host of antimicrobials are being used for growth promotion, treatment, and for prophylaxis; as a growth promoter, these antibiotics are used at sublethal or minute concentrations over a period, creating selective pressure for the development of antibiotic resistance [189,192]. The quantities of antibiotics employed in poultry farming vary with those used in piggeries and on cattle farms. In addition, geographical variation across regions and continents is observed in the antibiotics selected for use and the antimicrobial consumption patterns, affected by the animal species involved in food production, regional production patterns, types of production systems, intensive or extensive farming, the purpose of farming (industrial, commercial, or domestic farming), uncertain legislative polices relating to antimicrobial use, as well as the size and the socioeconomic status of the population and farmers in particular [213]. The use of antimicrobials in the livestock sector in low- and middle-income countries is increasing each day, causing a livestock revolution as the demand for animal food sources is rising alongside the farmers desiring quicker profits via increases in productivity [220].

Antimicrobial use in livestock causes bacteria colonising the animals to develop resistance owing to the selective evolutionary pressure of livestock antimicrobial use. This can be conveyed to humans via the consumption of animal products, direct contact, and environmental exposure. The World Health Organization [232] mentioned that the use of antimicrobials, regardless of the form, with inappropriate (minute) dosing for too short a period or the use of the wrong antibiotics can promote antimicrobial resistance. Accordingly, Darwish et al. [187] emphasised that the inappropriate use of antimicrobials and the disregard for observing the withdrawal period might result in the accumulation of antibiotic residues in animal tissues. Consistent exposure of humans to the antibiotic residues to significant levels via consumption of animal product can exert a negative impact on the microflora in the intestines, leading to the proliferation of antimicrobial-resistant bacteria. Infected animals can also become plausible reservoirs of antibiotic-resistant bacteria, which might eventually enter the food chain [40].

Antimicrobial resistance is a phenomenon wherein bacteria develop the ability to resist the effect of antibiotics which were previously effective in eradicating them or inhibiting their growth. This action currently threatens human health with a dark age as common minor infections could become potentially deadly. The WHO presents antimicrobial resistance as a major evolving challenge of global significance, threatening clinical, veterinary, and plant health [233]. Antibiotic resistance has a huge global impact on mortality, morbidity, and the economy, particularly in low- and middle-income countries, including South Africa. These countries are observing rapid population growth and urbanisation, encouraging the transmission of bacterial infections and easy access to antimicrobials, thereby promoting resistance. Several factors may be exacerbating the transmission of the antibiotic resistance genes between animals, humans, and the environment. 

South Africa has a great interest in livestock farming as the agricultural sector plays a key role in the development of the country’s socioeconomic capacity; therefore, almost three-quarters (70%) of its agricultural land is employed in livestock farming [234]. Livestock farming can take the form of intensive, communal, or rural farming depending on the population residing in a particular province, e.g., in Eastern Cape Province, the inhabitants rely on natural subsistence for livelihood [40]. In addition, individuals residing in both urban and rural areas of the country are involved in farming as they own and keep animals (cattle, sheep, goat, pigs, chickens) for several reasons, including for fertiliser production by using the manure, food, financial aid, social influence, etc. [235]. Livestock production systems create much greater contact between animals, humans, and the natural environment. Following metabolism, animals excrete antibiotics and antibiotic-resistant bacteria/genes in their urine and faeces into the environment. 

Zalewska et al. [236] pointed out that livestock farming is one of the most critical hotspots associated with the development and the propagation of genes associated with resistance to antibiotics as resistance genes have been isolated and identified from soil, animal faeces, animal housing, manure storage facilities, the areas around the farms and the guts of the farm animals. Manyi-Loh et al. [205] and Manyi-Loh et al. [204] recovered multidrug-resistant enteropathogens, including *Salmonella* spp., *E*. *coli*, *Campylobacter* spp., *Listeria* spp., and *Yersinia* spp., in cattle manure and pig manure, respectively, procured from animal farms in Eastern Cape Province of South Africa. In addition, Sithole and co-authors [192] in a farm-to-fork continuum in intensive pig production in the country demonstrated multidrug resistance in isolates of *C*. *jejuni* and *C*. *coli*, with the occurrence of *tet O*, *bla* _OXA-61_, and *cmeB* resistance genes, showing resistance to tetracycline and ampicillin as well as mutations in chromosomes occurring on *gyrA* (Thr-86-Ile) and *23S rRNA* (A2075G and A2074C) genes, exerting resistance to quinolone and erythromycin, respectively. Similarly, in a study conducted in KwaZulu-Natal Province, Pillay et al. [203] reported multidrug resistance in *Campylobacter* species recovered from intensive poultry farming, demonstrated by the presence of resistance genes, including *cmeB*, *tet O*, *gyrA* mutation, and A20175C/A2074G point mutation. 

In developing countries, including South Africa, people living in rural communities tend to have close contact with food animals, offering huge chances for the transmission of zoonotic bacterial pathogens [205]. Meat could be contaminated with microbes during slaughter and/or processing. The contaminating organisms are mainly derived from animal hide and faeces [237]. Therefore, humans living in proximity or close contact with animals kept for food are at risk of contact with bacteria that can cause infection. The pathogens can be transmitted via the faeces of the animals to humans in poor environmental conditions. Foodborne pathogens are considered as the principal source of infection in developing countries [237]. Animal and human interactions are increasing, presenting a great risk of zoonotic infections, and informal livestock trade is significant in South Africa [88], improving food security, reducing price instability, creating opportunities for jobs, as well as avoiding large multiregional foodborne outbreaks. The small-scale farmers in developing countries might not have the economic viability to procure advanced treatment options (e.g., sequencing batch reactors, trickling filters, or engineered wetlands) so inappropriate management and treatment of the wastes are bound to be employed [90].

Moreover, owing to the poor sanitation conditions reported in most low- and middle-income countries, waste management on some farms is deteriorating or substandard and animal manure is managed by dumping/piling in heaps in the open or applied as a biofertiliser to improve the growth conditions of crops [238]. Deliberate or unplanned release of manure into the environment via hydrologic processes (storms, heavy rainfall, leaching etc.) can cause antibiotic resistance genes and antibiotic-resistant bacteria to be released into underground springs and freshwater bodies, accumulating in the environment (animal and crops) and, eventually, transmitting to humans, leading to zoonotic infections [239].

Furthermore, limited access to veterinary services and the poor knowledge, attitude, and practice of the farm owners in relation to antibiotic usage and resistance can exacerbate the proliferation and transfer of resistance [48]. Several authors noted that poor awareness of antimicrobial resistance and the correct use of antimicrobials has been observed amongst several farm owners [240]; farmers and workers use antibiotics against viral infections and discontinue treatments as soon as the symptoms disappear. It is noted that the widespread and imprudent use of antimicrobials in food animals plays a remarkable role in the development of antibiotic resistance and antibiotic residues, presenting as a growing public and animal health concern in developing countries [240].

### The Role of Integrons in the Transmission of Antibiotic Resistance

*Enterobacteriaceae*, which are Gram-negative bacteria, exist as the natural flora in the gastrointestinal tract of both humans and animals. Bacteria belonging to the family *Enterobacteriaceae* are responsible for causing varied human infections, making treatment challenging owing to the emergence of multidrug resistance (MDR) to pan-drug resistance (PDR) [226]. The resistance can manifest owing to a mutation in a gene or by acquisition of resistance genes [241]. These resistance genes termed as mobile genetic elements (MGEs) can be chromosomal or plasmid-borne genes, including plasmids, integrons, and transposons. Integrons play a vital role in resistance transfer via the process of horizontal gene transfer by capturing, accumulating, and disseminating the genes through transmissible plasmids and transposons [242]. Horizontal gene transfer is recognised as the major cause of rapid multiplication of antibiotic resistance genes among widely diversified bacterial organisms [243]. Integrons as mobile genetic elements consist of unique backbone genes, enabling them via the process of homologous and non-homologous recombination to replicate the chromosome independently [244]. They are of a versatile structure accommodating several antibiotic resistance genes, therefore serving as a scaffold for the rearrangement of multiple genes by site-specific recombination gene cassettes. 

The mobile genes accommodated and rearranged in integrons are known as gene cassettes, carrying genes that are acquired, encoded, or deleted. These mobile genetic elements are amongst the factors responsible for the development of multidrug resistance with complex models through possession, expression, and dissemination of resistance genes [245]. Each integron harbours three fundamental components, including *int*I (integron integrase gene), *att*I (integron-associated recombination site), along with gene cassettes, and Pc/P*int*I (integron-associated promoter) [246]. Integrons can be differentiated based on mobility into two categories, namely, mobile integrons and super integrons. Shetty and colleagues [244] explained that mobile integrons rely on transposons or plasmids for movement and harbour antibiotic resistance genes with several *att*C sites but with only some gene cassettes. Sometimes, mobile integrons are known as resistant integrons or multidrug resistance integrons. On the other hand, Ravi et al. [247] described super integrons (chromosomal integrons) as having multiple gene cassettes, with multiple promoters with similar *attC* sites, and they are usually located on chromosomes. Recently, integrons were differentiated into five categories (integrons I–V) depending on the amino acid sequences of the integrase genes expressed as integrases (*intI*) 1–5. Initially, classes I–III integrons were associated with mobile gene elements (Table 3) whereas classes IV and V were found on chromosomes (but not extensively studied). All the different classes of integrons demonstrate varying potential in capturing gene cassettes, thus they behave differently in the dissemination of antimicrobial resistance whilst, amongst them, the class I integrons are more diverse, broadly distributed, and reported in animals and people plus demonstrate key roles in the spread of antimicrobial resistance. in*tI*1 and -2 are similar (about 46%), but the difference lies in the fact that *intI*2 is truncated early by the end codon (TAA), causing it to be weaker in moving the gene cassette, and demonstrates less variability than *intI*1. Likewise, *intI*1 and *intI*3 are similar, bearing four genes, including q*acEΔ1*, *orf5*, *orf6*, and *sul1*, but differ in that *intI*3 lacks transcription genes. Members harbouring the same class of integrons carry the same integrase but can have different gene cassettes [241]. The genes harboured in the cassettes can code for manifold genes, including the antibiotic resistance genes expressing resistance to chloramphenicol, trimethoprim, erythromycin, streptothricin, lincomycin, fosfomycin, quinolones, all aminoglycosides, all beta-lactams, rifampicin, and ammonium quaternary compounds. 

With emphasis, the integration of the environment, animal health, agricultural field, and public health can give a clear understanding of the global challenge of antimicrobial resistance. The circulation of resistant bacteria and antibiotic resistance genes among the described compartments is the cause of the emerging public and animal health-related threats. The integrons in bacteria facilitate the spread of antibiotic resistance genes between bacteria and from bacteria to humans either in the environment or via the food chain by the process of horizontal/lateral gene transfer. The integron is simple but compact and harbours DNA elements with open reading frames and recombination sites (*attC*) [242]. Through the process of reversible cassette-associated recombination, integrons can capture new genes, resulting in subsequent evolution, wherein new genetic materials are integrated into the bacteria via site-specific recombination (*attI*) and expressed by the same promoter of the host bacterial genome. Integron integrase facilitates the integration of the gene cassettes into *attI* and *attC*. The recombination site (*attC*) produces important secondary stable structures responsible for the identification of *intI* along with recombination [248]. Integrons contribute to the evolution of resistance owing to their rapidly generated combinatorial variation in the composition of the cassette while maintaining the integrity of the genome [249].

## 4. Public Health Implications of Consuming Microbial–Antibiotic-Contaminated Meat

Meat is a highly nutritious food from which several other products (sausages, hamburgers, polony, etc.) are derived, playing an essential role in human nutrition; therefore, its hygienic value is paramount for public health, considering that consuming poor-quality meat and its products can lead to infections and other adverse health effects [17]. Of major concern to public health is the outbreak of foodborne diseases, owing to their rising incidence globally and great burden of mortality and morbidity caused by bacterial infections. The consumption of contaminated food, especially animal products, including diseased carcasses or food contaminated with pathogenic microbes, usually results in foodborne illness, which exerts a serious socioeconomic impact in most developing countries [108]. Meat is the most important source of antibiotics. Ingestion of antibiotic residues through animal products negatively affects human health, causing hypersensitivity reactions (allergies), hepatotoxicity, teratogenicity, reproductive disorders, mutagenicity, carcinogenicity, destruction of the normal and or useful intestinal flora, as well as indigestion [186,189]. Consequently, withdrawal periods and maximum residue limits (MRLs) are instituted in food safety legislation in a bid to lessen the presence of these antimicrobial drugs in animal-derived products [250]. Mandatorily, a waiting or withdrawal time and an analysis of the physicochemical parameters of meat are carried out to make sure the concentration of the antibiotics used, or their analogs, does not exceed the maximum residual limit, prior to marketing. The authors further explained that on a global scale, animal husbandry is a key feature of the economy in addition to being the main contributor to food provision; therefore, for the animals to acquire weight, they are fed with feed containing antibiotics or are given water containing antibiotics for drinking. Nevertheless, antibiotics are also mixed with water and administered to the animals for preventive purposes [156]. 

Contamination of meat or meat products can occur from many sources and the level of contamination of meat with microbes and its floral composition reflect the standard hygienic condition of the meat. Meat and its products are the major reservoirs of *S*. *aureus, Campylobacter* spp., etc. Tshipamba et al. [85] investigated the occurrence of bacterial pathogens in ready-to-eat meats in the Johannesburg area; the authors noted a prevalence of 25% of *S*. *aureus* amongst the fifteen bacterial pathogens (*Enterococcus faecalis*, *Planomicrobium glaciei*, amongst others) that were identified in 115 samples. Similarly, Ijabdeniyi et al. [251] registered a higher prevalence of *S*. *aureus* amongst *L. monocytogenes*, *Salmonella, Enterobacter cloacae*, *Citrobacter freundii*, and *Lelliottia amnigena* recovered from ready-to-eat meat procured from households and retail locations in Durban and a higher prevalence in the household samples as opposed to the retail samples. Meat must be properly cooked before it is consumed by humans and adequate thermal application during processing takes care of the vegetative forms of some bacteria, including *S. aureus* [10]. However, the degree of cooking varies by the individual and inadequate supply of heat causes some bacteria to remain active and some enterotoxins cannot be destroyed by heat as they are thermostable and have demonstrated resistance to proteases occurring in the gastrointestinal tract (e.g., *S*. *aureus*). 

It is ascertained that the health of the population is greatly influenced by the food they eat and the environment in which they reside. The detection of bacterial pathogens in meat not only indicates their ability to disseminate antibiotic resistance but reveals the potential to cause serious human infections, including septicaemia, infection of the urinary tract, and pyogenic infections, amongst others [57]. 

Owing to increased demand for meat, farmers responded with the implementation of excessive antimicrobials as the most accessible and immediate approach to increase the production of livestock and poultry meat, thereby addressing the demand and preventing the spread of diseases in the animals [204]. The interplay of microorganisms from nature, hospitals, and livestock facilities could cause the bacteria to evolve resistance to multiple antibiotics, which can severely affect environments and can cause the alteration of biodiversity and evolution paths in favour of resistance [252]. The resistance in the pathogens could be primary (the infecting bacterium becomes resistant at the outset) or secondary (when the pathogen becomes resistant during treatment due to non-compliance to the antibiotic treatment regimen or inappropriate use of antibiotics) [253]. Over the years, microorganisms have developed resistance against antibiotics that they used to be sensitive to, thus they were used in the treatment of the infections caused by the bacteria. Recently, a plethora of newer generation antibiotics and pharmaceuticals of last resort have become ineffective against bacteria that were formerly termed as susceptible, thus causing a huge challenge for health care managers worldwide, especially as there is a discovery void in the development of new antibiotics [254]. According to Jamrozik and Selgelid [255], the drug-resistant pathogens originate, spread, and exert adverse effects based on several factors, including biological processes (e.g., microbial evolution, distribution of resistance genes between microbes, and immunity of human host), human behaviours (e.g., antimicrobial use and hygienic practices), and social factors (e.g., access to clean water, sanitation, health care, and antimicrobials). Overall, the implications of drug-resistant pathogens can be categorised into direct effects on human beings (health and health care), economic effects, and burdensome public health interventions. Nevertheless, the undesirable effects of antibiotic resistance produce varying impacts between regions and countries on human health and food safety, as it is affected by the interaction of some predisposing factors such as land use, human population, animal demography, national policies, contaminated water supplies, and national and international trade. Equally, antibiotic resistance is distributed via intensive food production, globalisation of food distribution, international travel, changing climate, increased population growth, and urbanisation [156]. 

The singular threat arising from antibiotic-resistant bacteria (ARB) is the acquisition of MDR, XDR, and PDR [41]. In a farm-to-fork study performed in an intensive poultry farming system, in KwaZulu-Natal Province, Pillay and colleagues [203] registered the occurrence of multidrug-resistant *Campylobacter* isolates, posing a threat to the safety of food. Likewise, Sithole et al. [192] noted a great prevalence of multidrug-resistant *Campylobacter* isolates (87.3%) in samples obtained from the farm, transport, abattoir, and retail pork using a farm-to-fork sampling approach. *C. jejuni* and *C. coli* are zoonotic pathogens incriminated as the cause of campylobacteriosis in humans. Considering the high intake of meat products by the South African population, we cannot rule out the fact that everyone in the country is directly or indirectly connected to the production, processing, and consumption of meat products. It is a more disturbing factor regarding the population of South Africa when considering that a great fraction of this population is HIV/AIDS-positive, who depend on antibiotics to enhance their immune systems while managing several infections, including gastrointestinal infections that are known to be recurrent in this category of people [256]. Drug-resistant pathogens have a serious long-term effect on health systems alongside serious and instant effects on those affected [253]. Globally, the health of the population is currently affected by widespread bacterial infections caused by bacteria expressing decreased sensitivity to the highly recommended drugs employed for antimicrobial chemotherapy. Therefore, after failed treatment, the patient’s condition deteriorates (because of increased side effects resulting from multiple and powerful drug use), requiring the use of either second or last line antibiotics, thus creating a treatment regimen with antibiotics that are very costly, less available, and associated with severe side effects [257]. In addition, adverse effects on the pharmaceutical industry and society at large as well as elevated financial constraints on the people and the facility engaged in the delivery of health care services occur [8]. 

More elaborately, the antibiotic-resistant pathogens demonstrate high occurrence across the globe, presenting as a silent pandemic in public health as the therapeutic options for infections caused by this group of bacteria are limited, therefore significant morbidity and mortality become inevitable with huge monetary impact [258]. Consequently, antibiotic resistance has the potential to even reverse the gains made in public health [254]. The transmission or acquisition of resistance genes can further potentiate the pathogenicity of a bacterium, making it more virulent. Interestingly, Beceiro et al. [259] affirmed an association between resistance and virulence which may be beneficial to pathogenic bacteria. The authors further revealed that resistance and virulence factors have common characteristics, including being needed by the pathogenic bacteria to evade the host defence mechanisms in order to survive, being transferred from species to species or between genera via horizontal gene transfer, antibiotic resistance usually being connected with infection, as is virulence, and both demonstrating direct involvement with efflux pumps, porins, cell wall alterations, and two-component systems that activate or repress the expression of various genes. Antimicrobial resistances can heighten the virulence or fitness of some species in certain environments, thereby helping these species to colonise new niches. However, in certain situations, antibiotic resistance is a crucial factor in the development of infection, and it may be considered a virulence-like factor in specific ecological niches which antibiotic-resistant bacteria are able to colonise. Resistance and virulence can act as a deadly duo as many infections are becoming highly untreatable and uncontrollable, especially when the antibiotic pipeline is extremely dry, therefore facilitating the likelihood of reverting to the pre-antibiotic era. This is a fearful situation that might cause a therapeutic dead end in the treatment of infections caused by bacteria. Untreatable infections not only cause mortality and morbidity but can equally exert broader consequences on human freedom, privacy, and wellbeing [255]. Of more concern is that no new antibiotics are being discovered because small pharmaceutical companies might not be financially able to afford the cost of carrying out complex clinical trials involving antibiotics. Therefore, companies shift their attention towards the production of medications used in treating chronic illness (diabetes and hypertension), because no resistance is built even though the patients are taking the drugs over a prolonged period; this appears as a more lucrative business proposition to these companies [254].

The presence of vancomycin-resistant enterococci, methicillin-resistant staphylococci, extended beta-lactamase-producing *Enterobacteriaceae*, carbapenemase-producing *Enterobacteriaceae* and Gram-negative bacteria, carbapenem-resistant *Klebsiella*, and the pan-drug-resistant *Pseudomonas* and *Acinetobacter* are causing difficult-to-treat infections, creating havoc in the treatment regimens in health facilities across the globe and resulting in deaths and delay in healing [254]. The authors further reiterated that the occurrence of resistant pathogens has a tremendous negative effect on public health, hampering the control of infectious diseases because the delay in healing causes the patient to be infectious for a longer period, enhancing the chances and risk of transmission of resistant pathogens to others. Therefore, the patient is a reservoir of infections for a longer time, endangering the lives of more members of the community and workers in health care facilities. Hospital- and community-acquired resistance contributes to a chronic burden of disease [253]. It is believed that with changing lifestyles and an ageing population, the chronic diseases that are currently treated via surgery will increase and this would be impossible without the availability of effective antibiotics because these antibiotics have an inseparable link with certain medical procedures, including heart surgery, post-organ transplantation, diabetes-related chronic infections, and aggressive immune-modulating therapy for autoimmune diseases (e.g., rheumatoid arthritis). On the other hand, for farmers and the food industry, food production can be negatively affected due to the lack of effective antibiotics to treat sick animals. Also, farmers are at higher risk of exposure owing to their close contact with animals that might be colonised with resistant pathogens [260].

Naturally, there exists a balance in the bacterial strains inhabiting the gastrointestinal tract, comprising 95% beneficial strains and the remaining portion comprising opportunistic bacteria. The consumption of antibiotics alters the composition (creating imbalances) of the bacterial strains in the gut, resulting in antibiotic-resistant bacteria/pathogens and the proliferation of opportunistic bacteria. The emergence of antibiotic resistance in Gram-negative *Enterobacteriaceae* and the genera *Pseudomonas* and *Acinetobacter* is of high consideration and commands great attention according to the World Health Organization. It explains that these bacteria produce extended beta-lactamases conferring resistance to a few antimicrobials, constituting all the new generation antimicrobials that are used as the last line of antibiotics for defence against resistant pathogens [261]. Therefore, drug-resistant pathogens compromise the treatment of infections, thereby undermining numerous developments in surgery, cancer treatment, and immunosuppression that rely on our capacity to effectively treat infections [255]. Some species of these bacteria demonstrate broad niche colonisation (widely distributed), and their growth can be enhanced in a contaminated environment; some of the species are opportunistic in nature, able to cause infections in sick or immunocompromised people and they are linked with hospital-acquired infection, sepsis, secondary pneumonia, etc. Consequently, alterations can lead to certain conditions, including pseudomembranous colitis, colorectal cancer, and intestinal disorders. In addition, the imbalance in the gut bacteria can dysregulate development of the immune system, leading to adiposity and bone growth in the early stage of life [262]. In conclusion, antibiotic resistance is of global concern to both humans and animals and requires collective management as no country or discipline exists in isolation [51]; countries interact through travelling, exports, and trade.

## 5. Ways of Controlling Contamination with Resistant Pathogens

Animal husbandry serves as a platform via which milk, eggs, and wool are provided to the human population, therefore it presents as a fundamental part of the agricultural economy and performs a key role in supporting the livelihood of those living in rural settings. However, the agricultural setting is considered as a scenario for the generation of antibiotic resistance owing to the huge quantities of antibiotic employed. Several findings noted methicillin-resistant *S*. *aureus*, vancomycin-resistant enterococci, extended-spectrum beta-lactamase-producing bacteria, etc. [263]. Recently, there has been a rise in the development and distribution of antimicrobial resistance or drug resistance, whereby it presents as a global challenge and is extremely dangerous. Nevertheless, the drug resistance differs in different bacteria across the different regions, nations, and countries of the world. The dissemination of zoonotic pathogens and their drug-resistant counterparts can occur via numerous means, including consumption of contaminated water or food (milk, eggs, meat, protein, etc.) plus direct contact with animals. However, the food chain appears to be the most probable route of transmission [229]. Apparently, identifying the sources and origins of drug-resistant bacteria is a prerequisite necessary in the control of contamination with resistant bacteria via reducing their transmission [222]. MacIntyre and Bui [253] explained that the understanding of epidemiology (prevalence/incidence, mode of transmission, distribution etc.) of AMR is a paramount step in its control. In addition, microorganisms are developing resistance at a much faster pace which is not reconciled with the production of new antibiotics. Therefore, the need for the control of these drug-resistant bacteria becomes obvious. The control strategies are reservoir-based or transmission-based. Generally, control can equally target the spread or transmission, wherein the spread of the drug-resistant bacteria could be limited via employing antimicrobial stewardship, especially in developing countries, engaging in the production of novel antimicrobials, improvement of practices along the food production chain, reducing the quantities of antibiotics sold for use in animals, as well as improvement of antimicrobial resistance surveillance programmes [264]. 

One Health approach: According to Akinsuyi and colleagues [265], the One Health approach is defined as a unique approach which is multisectoral, transdisciplinary, and collaborative and can determine the human–animal–environment transmission, creating great opportunities to tackle and reduce the prevalence of these bacteria by curbing their spread. This is because the One Health concept recognises the interconnectedness between human and animal health in association with the ecosystem where they live as well as endorses that the sustained growth of the human population is influenced by climate change and the decrease in natural resources, so several disciplines (public health, epidemiology, microbiology, sociology, agriculture) work together for the security of the health of humans, animals, and ecosystems at the global level [266]. This can be achieved through reducing the quantities of antibiotics employed in animal farming as well as enforcing rational use of the antibiotics (minimising the purchase of over-the-counter antibiotics and providing access to quality and affordable drugs) [258]. Antibiotic resistance is associated with antibiotic usage, therefore reducing the amount of antibiotic use will result in the simultaneous reduction in the likelihood of generation of antibiotic resistance in the bacterial population [267]. It is explained that the elevated concentrations of antibiotics in the environment cause selective pressure, altering the genetic makeup of native environmental bacteria predisposed to the development of antibiotic resistance [268].

In addition, the bodies of animals do not completely metabolise the antibiotics administered to them whether for growth promotion, treatment, metaphylaxis, or prophylaxis [229]. Thus, the unmetabolised portion of the antibiotics is excreted alongside resistant genes and bacteria into their wastes (e.g., urine and faeces). The wastes may eventually spread their components via fertilisation with raw manure or hydrological processes of storms and heavy downfall into water bodies or surface runoff, polluting the environment [269]. Therefore, appropriate collection, management, and treatment of these wastes will help in curtailing their transfer and prevent them from ultimately ending up in the environment. This is possible via the use of anaerobic biodigesters to degrade the wastes into methane and biofertiliser. Manyi-Loh et al. [204] demonstrated the reduction of drug-resistant strains of *E*. *coli*, *Salmonella*, *Campylobacteria* spp., and *Yersinia enterocolitica* present in pig manure over time during anaerobic co-digestion with lignocellulosic waste (sawdust). 

Surveillance, monitoring, and research: Owing to the diversity and geographical variation in drug sensitivity patterns and bacterial spectra, local, regional, national, and international programmes on AMR surveillance are pertinent [223]. Moreover, establishing databases of drug-resistant bacteria in humans, animals, and the environment at the local, regional, and national level will give clear indications of the true burden of drug-resistant bacteria as well as identify potential ways to curb them [265]. There is a lack of an in-depth comprehension of the epidemiology of resistant pathogens, therefore improvement in local and international public health surveillance would aid in determining the impact of the different mechanisms of resistance as well as evaluating the cost-effectiveness of the interventions [255]. Consistent and routine investigation into identifying the different sources and types of resistant bacteria and their resistance genes is of great human and public health significance so as to assemble information on the current antibiotic resistance trends and profiles, thus updating the local, regional, national, and international databases and strengthening knowledge [221]. Individuals as well as communities vary in their languages and perceptions relating to antimicrobial use and antimicrobial resistance. Enforcing sensitisation campaigns throughout the community (community engagement) about the awareness of antibiotic resistance and harnessing the community’s potential via active community engagement and involvement from the design to the implementation of the chosen interventions will go a long way in combating drug resistance [221]. Research focusing on integration strategies, which include the development of vaccines, diagnostics, and new antibiotics to combat AMR through collaborative efforts of academia and industries both at the national and international level, is significant [270]. Vaccines have been employed for a long time as a prophylactic measure in the prevention of infectious diseases, thus limiting the use of antibiotics for treatment purposes or precluding the need to prescribe antimicrobials for an array of pathogens [258]. The production of effective vaccines against viral infections can equally lead to a reduction in the use of antibiotics because some viral infections are erroneously treated with antibiotics. In the same vein, investigations into identifying suitable alternatives to antibiotics should be performed and these include plant-based phytochemicals, bacteriocins, and antivirulence agents that can be employed as antibacterial therapy. Furthermore, Steffan and co-authors [117] mentioned that bacteriophages are encouraging in the aspect of reducing resistant bacteria in food production plants, describing phages as natural predators of bacteria. 

Infection prevention and control/sanitation measures: Along the food production chain, good or strict hygienic practices, adequate animal housing, and the presence of proper animal health management avoid or reduce infections, avoiding the use of antibiotics as growth promoters or reducing their demand for treatment [271]. Infections caused by drug-resistant pathogens can be avoided via the implementation of an infection prevention and control (IPC) approach, an essential and evidence-based strategy to safeguard patients and health workers from being affected. The IPC entails complying with hospital infection control and antibiotic policies and the judicious reporting to the IPC team about resistant cases, education of health care workers and nurses about AMR and aseptic procedures necessary in the control of infections, as well as sensitising patients about compliance to treatment, especially antibiotics that provoke the development of resistance, which should be administered by physicians, nurses, and other health workers and pharmacists in order to combat AMR [258]. In addition, Samreen and colleagues [270] remarked that access to clean water will enhance hygiene and sanitation, limiting the spread of drug-resistant pathogens.

Antimicrobial stewardship programme: Antimicrobial stewardship is implemented to ensure the prudent use of antibiotics, operating to optimise therapy and minimise the consequences of antibiotic resistance because it considers the selection, dosage, and the duration of the antimicrobial therapy during the period of use [272]. In this light, microbiology laboratories are necessary for the accurate detection of disease-causing agents in animals and profiling their sensitivity to conventional antibiotics. Therefore, capacity building of diagnostic facilities will facilitate antimicrobial stewardship [267]. A successful antimicrobial stewardship takes a multidisciplinary approach involving the expertise of ICU physicians, infectious disease physicians, microbiologists, and pharmacists and is anchored on several key elements, including pre-authorisation, prospective auditing and feedback, facility-specific treatment guidelines, antibiotic time outs, pharmacy-based interventions, rapid diagnostics, and clinical decision support systems [228]. Antimicrobial stewardship helps in improving the clinical outcome of patients as well as avoiding the overuse of antibiotics, and it is effective in reducing the burden of resistant bacteria. Equally significant is the development of novel antibiotics to target the resistant bacteria. Abbas et al. [273] recommended that strict guidelines associated with antibiotic use should be instituted and they must be implemented by hospital administrations and policy makers. The authors further remarked that all patients should be subjected to culture and sensitivity testing prior to antibiotic administration. This indicates the significance of *in vitro* susceptibility in a bid to avert the adverse effects associated with delayed therapy, including marked increases in morbidity, mortality, overall cost of treatment, the need for surgical intervention, and length of hospital stay [274]. Furthermore, having knowledge of the *in vitro* susceptibility of the causative bacterium to antibiotics will reduce the use of broad-spectrum antibiotics, reducing the selection of resistant strains as well as reducing their numbers, thus leading to their reduced chances of transmission [275]. The suitable use of antimicrobials in both humans and food-producing animals will go a long way in maintaining the effectiveness of the drugs, while reducing the chances of spread of antibiotic resistance. This is backed by the fact that it is the misuse or overuse of antibiotics that brings about resistance [260]. 

## 6. Conclusions

The safety of meat is challenged by invisible biological and chemical contaminants, including microorganisms plus their toxins, residues of veterinary drugs, and environmental contaminants that do not alter the physical appearance of the meat or do not cause changes in the animal’s organs or cause the animal to present with any clinical symptoms (i.e., the animal appears asymptomatic) [276]. South Africa is recognised as a key contributor to the total increase in meat intake at the global scale. The meat becomes vulnerable through handling, manipulating, processing, and packaging occurring in the food supply chain until it reaches the end users (consumers). Therefore, heightening of hygiene and sanitation practices plus implementing other methods (during transportation, slaughtering, processing of meat and meat products) aimed at preventing bacterial entry and proliferation in food as well as preventing contamination with antibiotic resistance genes are very crucial. The excess application of antibiotics in livestock and poultry farming has increased the incidence of antibiotic resistance globally because of the emergence of antibiotic-resistant bacteria as well as the transfer of antibiotic resistance genes between species via horizontal gene transfer. The resistance, which could be primary or secondary, can be expressed either as MDR, XDR, or PDR. The profiles of drug-resistant bacteria vary across the regions of South Africa, creating an impact on the health of the population as well as socioeconomic consequences. This is a concern because a large fraction of the South African population consists of immunocompromised individuals. Therefore, the One Health approach for mitigating the resistance to antimicrobials from human, environmental (food, plants, soil, water, etc.), and animal perspectives becomes crucial. This will drastically curtail the devastating effects on public health owing to the emergence or re-emergence of zoonotic diseases caused by the variability at the human–animal–environment interface. 

## 7. Future Directions

It is established that the use of antibiotics for the prevention of infection and growth promotion in animal food production precedes the development of antibiotic resistance. Antimicrobial resistance is a great menace to the sustainable production of animal protein in the long term to meet the ever-rising demand by the population. It is anticipated that a future reduction can occur in antibiotic resistance via lessening the consumption of antibiotics during animal farming. The ideology suggests that reduced quantities of antibiotics will be employed. Therefore, a surveillance system on antibiotic use in animal farming should be strengthened in a bid to gather data on the volume consumed, which will give an indication of the burden on the environment and human health [277]. The data will equally give a trend in antibiotic consumption resulting in monitoring which will ultimately express the evidence or the extent to which resistance due to antimicrobial use is addressed. Moreover, robust surveillance on antimicrobial resistance should be implemented to monitor the progress or the efficacy of the intervention measures operated in a bid to reduce antimicrobial resistance [278]. The authors proclaimed further that the reasonable and discrete use of antimicrobials plus the prevention of diseases in animal farming are key routes to lessen antimicrobial use. Accordingly, the prudent use of antimicrobials involves management and treatment options that replace or do not need the use of antimicrobials. Furthermore, detailed research into the numerous organic ways and alternatives to improve animal health and agriculture and augment food products should be investigated and encouraged [279]. Doidge and colleagues [280] highlighted the dire need for social science studies to sustain effective implementation. In addition, an improvement in the hygienic and sanitation on farms can reduce the risk of infections [213]. 

## Figures and Tables

**Table 2:** Prevalence of multidrug-resistant bacterial pathogens recovered from animal farming in South Africa.

Organisms	Prevalence of MDR (%)	Sample Sources	Types of Resistance Genes	Provinces	References
*Campylocater coli, Campylobacter jejuni,* *Campylobacter fetus*	ND	Retailed raw meat	*cat* II, *tet* A, *gyr* A, *amp* C, *aac*(3)-IIa(*aacC*^2^) ^a^, *tet* M, *erm* B, *tet* B, *tet* K	Eastern Cape	Igwaran and Okoh [109]
*E*. *coli*	39.08	Fresh pork meat	*Mrc*-1, *erm* B, bla *_TEM_*	Eastern Cape	Iweriebor et al. [193]
*Stapylococcus aureus, Enterococcus faecalis*, *Planomicrobium glacei*	53.33	Chicken, beef, intestines, beef head, and chicken gizzards	ND	Gauteng (Johannesburg)	Tshipamba et al. [85]
*Salmonella* spp.	43	Faeces from chicken, ducks, cows, pigs, goats, and sheep	*bla _TEM_*, *bla*_CMY-2_, *sul* 2, *tet* C, *dfr*A7, *tet* A	KwaZulu-Natal	Mthembu et al. [194]
*Campylobacter* spp.	87.3	Farm-to-fork scheme of intensive pig production. Samples include whole cuts (head, body, thigh, post-slaughter abattoir), carcass swabs, caecal samples, and rinsed carcass. Hand and nasal samples, faeces, litter, and slurry	*Tet* O, *bla _OXA-61_*, *cme*B, *gyr*A (Thr-86-IIe), A2075G/A2074C	KwaZulu Natal	Sithole et al. [192]
*Salmonella (Enteritidis*, *Hadar*, *Heidelberg*, *Stanley*)	20	Raw intact beef cuts, kidneys, intestines, tripe, liver, lungs, spleen, processed beef product	ND	KwaZulu Natal	Naidoo et al. [60]
*Salmonella enterica*	ND	Sheep, cattle, pig meat	*aad*A, *aacC*2, *aph* A1, *aph*A2, *Str*A, *amp* C*, bla _TEM_*, *bla _Z_*, *bla _OXA_*, *cat*I, *Cat*II, *tet* (A, B, C, D, K, M), *sul*I, *sul*II	Eastern Cape	Jaja et al. [195]
*Salmonella* spp.	3.8	Farm-to-fork approach in poultry (as mentioned above)	ND	KwaZulu-Natal	Ramtahal et al. [39]
Diarrhoeagenic *E*. *coli*	73	Farm-to-fork approach in pig farming	ND	KwaZulu-Natal	Abdalla et al. [196]
Listeria *monocytogenes*	76–100	Meat, milk, vegetables, water	ND	North West	Tchatchouang et al. [178]
*Listeria* spp.	100	River and irrigation water	*Sul*I, *bla _TEM_*, *tet*A, *bla_CIT_*	Eastern Cape	Mpondo et al. [197]
*Listeria* spp.	85.71	Milk	*bla _TE_*_M_, *bla _SHV_*, *bla _TEM_* variants (TEM-1 and TEM-2), *bla* Z, *tet* A, *Tet* D, *Tet* G, *tet* M, *tet* K, *aph*(3)-IIa(*aph*A2)a	Eastern Cape	Kayode and Okoh [198]
*Listeria* spp. *Aeromonas* spp.	100	Rivers and effluents from wastewater treatment plants	ND	KwaZulu-Natal	Olaniran et al. [199]
*Salmonella* spp. *S*. *aureus*	Approximately 100	Broiler chickens	ND	KwaZulu-Natal	Mkize [200]
*Escherichia coli*	32.7	Discharged final effluent of wastewater treatment	*Str*A, *aad*A, *cat*I, c*ml*AI, *bla _TEM_*, *tet* A, t*et* B, *tet* C, *tet* D, *tet* K, *tet* M	Eastern Cape	Adefisoye and Okoh [201]
*Listeria* spp.	41.86	Pig manure plus pinewood sawdust	ND	Eastern Cape	Manyi-Loh et al. [180]
*Escherichia coli*	80	River	ND	Western Cape	Lamprecht et al. [202]
*Campylobacter coli* *Campylobacter jejuni*	73.33 26.67	Farm-to-fork approach in poultry farming	The gyr A mutation, A20175C/A2074G point mutation, tetO, cmeB	KwaZulu-Natal	Pillay et al. [203]
*E. coli*	91.19	Pig manure plus pinewood sawdust	ND	Eastern Cape	Manyi-Loh et al. [204]
*Yersinia* sp.					
*Campylobacter* sp.					
*Salmonella*					
*Salmonella* sp.	48.19	Cattle manure	ND	Eastern Cape	Manyi-Loh et al. [205]
*Campylobacter jejuni*					
*Campylobacter* spp.					
*Escherichia. coli*					
*Shigella* sp.					

MDR, multidrug resistance; ND, not done; ^a^, alternative nomenclatures are in parentheses; acc(3)-IIa(aacC2), plasmid encoded aminoglycoside acetyl transferase; *cat*, chloramphenicol acetyl transferase; *tet*, tetracycline resistance gene; *erm B*, erythromycin resistance methylase gene; *gyrA*, DNA gyrase gene; *amp C*, group I cephalosporinase; *acc*(3), aminoglycoside N-acetyl transferase; *aad*, aminoglycoside adenyl transferase; *bla _TEM_*, extended-spectrum beta-lactamase; *aph*, aminoglycoside phosphotransferase; *str*A, streptomycin resistance gene; *Mcr*, mobilise colistin resistance gene; *sul*I, sulfonamide resistance gene; *dfr* A, dihydrofolate reductase; *cme*B, chloramphenicol efflux transporter; *cml*, chloramphenicol resistance gene.

**Table 3:** Different classes of integrons and some of their gene cassettes expressing antibiotic resistance (adopted and modified from Sabbagh et al. [241] and Shetty et al. [244]).

Integron	Gene Cassettes Linked with It	Description of the Gene Cassette	Antibiotics Involved in the Resistance	Bacteria Harbouring the Gene Cassettes
Class I	*Sul*1, *qac*EΔ1, *drf*A, *cat*, *aad*A, *rpo*, *aad*A1, *dfr*A12-gcuF-*aad*A2, *aad*A1a, *drf*A17, *bla* _CARB-2_, *dfr*A17-*aad*A5, *dfr*A12-*orfF*-*aad*A2, *aacA*4-*cml*A1, *tet*, *ere* 2, *aad*B, *aad*A5, *dfr*A12, *drf* A1-*aad*A1, *bla _OXA-101_*, etc.	Dihydrofolate reductase, aminoglycoside adenyl transferase, extended-spectrum β-lactamase, tetracycline resistance protein, chloramphenicol efflux transporter, sulfonamide resistance genes	Sulfonamides, quatenary ammonium compounds, trimethoprim, chloramphenicol, aminoglycosides, carbenicillin tetracyclines, macrolides, beta-lactams	*E. coli, Salmonella, Shigella, Klebsiella, Streptococcus, Staphylococcus, Enterobacter*
Class II	*dfr*A1-*sat*1-*aad*A1, *dfr*A1, *sat*1, *aad*A1, *ere*A, *dfr*A1-*sat*2-*aad*A1, *est*X-*sat*2-*aad*A1, d*rf*A14, *sat*2-*aad*B-*cat*B2, etc.	Streptothricin-acetyl transferase, aminoglycoside adenyl transferase, erythromycin esterase, chloramphenicol acteyltransferase	Trimethoprim, streptomycin, erythromycin, streptomycin/spectomycin	*P. aeruginosa, E. faecalis, E. coli*
Class III	*qacE*Δ1, *sul* 1, *orf*5, *orf*6, *bla* _IMP-1,_ *aac*A4, *bla*_GES-1_, etc.	Metallo-lactamase, sulphate permease, extended β-lactamase, quaternary ammonium compound efflux SMR transporter QacE delta1	Quaternary ammonium compounds, sulfonamides, beta-lactams, aminoglycosides, puromycin	*Serratia marcescens, E. coli, Pseudomonas putida, K. pneumoniae, P. aeruginosa, Salmonella* spp., *Citrobacter freundii*

## Data Availability

Not Applicable.
